# Precision drug design against *Acidovorax oryzae*: leveraging bioinformatics to combat rice brown stripe disease

**DOI:** 10.3389/fcimb.2023.1225285

**Published:** 2023-10-11

**Authors:** Arif Ali Khattak, Jiahui Qian, Lihui Xu, Ali Athafah Tomah, Ezzeldin Ibrahim, Muhammad Zafar Irshad Khan, Temoor Ahmed, Ashraf Atef Hatamleh, Munirah Abdullah Al-Dosary, Hayssam M. Ali, Bin Li

**Affiliations:** ^1^ State Key Laboratory of Rice Biology and Breeding, Key Laboratory of Molecular Biology of Crop Pathogens and Insects, Institute of Biotechnology, Zhejiang University, Hangzhou, China; ^2^ Institute of Eco-Environmental Protection, Shanghai Academy of Agricultural Sciences, Shanghai, China; ^3^ Plant Protection, College of Agriculture, University of Misan, AL-Amarah, Iraq; ^4^ Department of Vegetable Diseases Research, Plant Pathology Research Institute, Agriculture Research Centre, Giza, Egypt; ^5^ College of Pharmaceutical Sciences, Zhejiang University, Hangzhou, Zhejiang, China; ^6^ Xianghu Laboratory, Hangzhou, China; ^7^ Department of Botany and Microbiology, College of Science, King Saud University, Riyadh, Saudi Arabia

**Keywords:** *A. oryzae*, bacterial brown stripe, protein-protein interaction, molecular docking, Enfumafungin, minimum inhibitory concentration

## Abstract

Bacterial brown stripe disease caused by *Acidovorax oryzae* is a major threat to crop yields, and the current reliance on pesticides for control is unsustainable due to environmental pollution and resistance. To address this, bacterial-based ligands have been explored as a potential treatment solution. In this study, we developed a protein–protein interaction (PPI) network for *A. oryzae* by utilizing shared differentially expressed genes (DEGs) and the STRING database. Using a maximal clique centrality (MCC) approach through CytoHubba and Network Analyzer, we identified hub genes within the PPI network. We then analyzed the genomic data of the top 10 proteins, and further narrowed them down to 2 proteins by utilizing betweenness, closeness, degree, and eigenvector studies. Finally, we used molecular docking to screen 100 compounds against the final two proteins (guaA and metG), and Enfumafungin was selected as a potential treatment for bacterial resistance caused by *A. oryzae* based on their binding affinity and interaction energy. Our approach demonstrates the potential of utilizing bioinformatics and molecular docking to identify novel drug candidates for precision treatment of bacterial brown stripe disease caused by *A. oryzae*, paving the way for more targeted and sustainable control strategies. The efficacy of Enfumafungin in inhibiting the growth of *A. oryzae* strain RS-1 was investigated through both computational and wet lab methods. The models of the protein were built using the Swiss model, and their accuracy was confirmed via a Ramachandran plot. Additionally, Enfumafungin demonstrated potent inhibitory action against the bacterial strain, with an MIC of 100 µg/mL, reducing OD_600_ values by up to 91%. The effectiveness of Enfumafungin was further evidenced through agar well diffusion assays, which exhibited the highest zone of inhibition at 1.42 cm when the concentration of Enfumafungin was at 100 µg/mL. Moreover, Enfumafungin was also able to effectively reduce the biofilm of *A. oryzae* RS-1 in a concentration-dependent manner. The swarming motility of *A. oryzae* RS-1 was also found to be significantly inhibited by Enfumafungin. Further validation through TEM observation revealed that bacterial cells exposed to Enfumafungin displayed mostly red fluorescence, indicating destruction of the bacterial cell membrane.

## 1 Introduction

Numerous crops are affected by the Gram-negative bacterium *Acidovorax oryzae*. Sugarcane, rice, maize, oats, sorghum, and millet are among the several crops impacted by this bacterium. Contaminated seeds are the most important source of inoculum and a mechanism for transmitting this bacterium to new plants and causing an outbreak (Song et al., 2004). Knowing the molecular basis for the infection of *A. oryzae* is important since bacterial brown stripe (BBS) has significant commercial value, due to which it has gained increasing attention in China (Xie et al., 2011; Li et al., 2015). Bactericidal agents are mostly used currently to prevent and manage bacterial infections. Owing to severe environmental pollution and bacterial resistance brought on by the excessive use of pesticides in countries that cultivate rice, it has become more important than ever to develop novel prevention and control strategies (Laxminarayan et al., 2013).

To address the challenges of drug resistance, alternative approaches such as drug repurposing have emerged as promising strategies (An et al., 2023). This method of drug repurposing mostly consists of computational strategies that are mostly based on transcriptional signatures, targets, networks, machine learning, structures using chemogenomics, and techniques in molecular docking (March-Vila et al., 2017). While drug repurposing has several advantages, it also has limitations that can affect its success. One such limitation is the unexpected outcome of interactions between the target and repurposed drug, often due to differences in the active sites where the drug acts. The structure–activity relationship may differ between the original and repurposed drugs due to variations in biochemical targets, rendering some repurposed drugs ineffective. However, the effectiveness of repurposed drugs has been demonstrated when used in combination with other medications, making this strategy a strong contender for leveraging the existing pharmacopeia for new therapeutic uses (Aubé, 2012).

The repurposing of drugs against bacteria through a network-based approach requires the protein interaction networks for these bacteria to be computationally analyzed (Pan et al., 2016). Because of the continuous spread of antimicrobial resistance, finding new potential therapeutic targets, which are based on their biological process, has become the need of the hour. In order to discover potential therapeutic targets, a comparative genomics study could be conducted using various genome database resources and software tools. These resources and tools can help identify genes and proteins that are crucial for the survival, growth, and important functions of pathogens within the host. By analyzing this information, researchers can gain insight into potential targets for therapeutic intervention. By conducting a comparative genomics study between host and pathogen, it is possible to filter out homologs in order to avoid potential toxic and side effects of newly designed drugs on the host. This approach can increase the success rate of drug design by ensuring that the drugs target the pathogen without harming the host (Sheikh, 2010; Sekyere and Asante, 2018).

Various circumstances where a pathogenic variant is associated with a disease in non-coding regions are difficult to target, which further limits the number of associations that are candidates for drugs development. Combining data from multiple biological networks and pathway databases can broaden the number of potential targets and increase association numbers that lead to effective treatments. Traditionally, target discovery has relied only on wet lab experiments, which involve highly expensive, unreliable, and time-consuming processes. With the development of bioinformatics and chemical informatics, multiple omics-based *in silico* therapeutic target discovery methods (computer-aided drug designing) have come to the fore (Katsila et al., 2016; Shanmugam and Jeon, 2017; Wooller et al., 2017). Integration of computer-aided therapeutic target discovery with big data containing omics-based data greatly shortens the time required for drug discovery and development cycle, and the scope of experimental targets, and minimizes the experimental cost (Dai and Zhao, 2015; Shanmugam and Jeon, 2017). The two main existing classes of ***in silico*** methods for potential therapeutic target identification are comparative genomics and network-based methods, while the important features that differentiate these two methods from one another are network-based approaches that can be used in infectious diseases as well as in non-infectious diseases. On the other hand, comparative genomics is only used for infectious diseases (Csermely et al., 2013; Rangel-Vega et al., 2015). However, these two different methods often complement each other in their advantages and disadvantages (Zhang et al., 2021). The ligand-based approach of drug repurposing uses a combination of the chemical moieties of individually approved and marketed drugs.

To address the unrelenting need for improved treatment alternatives for BBS disease, here we have used a combination of bacterial-based ligands, which were previously shown to be better inhibitors against multiple bacteria and fungus. In short, we have screened drugs using molecular docking, to propose Enfumafungin as a drug candidate against plausible bacterial resistance caused by *A. oryzae*. We are optimistic that using such molecular targets might help us overcome the rapidly growing antibiotic resistance in the *Oryza sativa*, with the help of rational development of effective drugs against BBS disease in the future. Furthermore, our combined-moiety ligand-based molecular docking approach via indispensable targets will likely provide new opportunities of drug repurposing for drug-resistant BBS.

## 2 Materials and methods

The genomic data and bacterial strain of *A. oryzae* were collected from Plant Bacteriology Laboratory, Zhejiang University, PR China. The bacterial strain was normally grown in Luria-Bertani (LB) medium consisting of 5 g of yeast extract, 10 g of NaCl, 10 g of tryptone, and 1,000 mL of ddH_2_O with/without 15 g to 20 g agar, pH 7.0, at 30°C as described by Masum et al. (2019).

### 2.1 Details about datasets and literature

One of the major bacterial pathogens of rice, *A. oryzae*, the causative agent of BBS, was selected in this study. The genomic data of the top 10 proteins were selected on the basis of genome analysis of the bacterial strain. Betweenness, closeness, degree, and eigenvector studies further narrow down our selection from 10 proteins to 2 proteins. The proteins we have chosen for our study were compared with the already available published data, and it was found that both proteins are crucial for conducting this study. Similarly, in order to confirm that the dataset we have chosen is valid or not, it was compared with the already available datasets and was found appropriate for our study. For further study, the dataset was filtered to find those with the least bias and noise. Both the case sample and control sample were present in the datasets and logarithmic modification was performed to reduce the impact of outliers and approximate normalcy.

### 2.2 Protein–protein interaction network analysis

Using all the shared differentially expressed genes (DEGs) of the *A. oryzae*, a protein–protein interaction (PPI) network was developed with the help of the STRING database (Szklarczyk et al., 2016). Setting up a pathway generally starts with a signaling molecule stimulating a specific receptor molecule, which further initiates a cascade of PPIs. Understanding this PPI is considered as a prerequisite for drug development and systems biology. The study of PPI networks helps us understand a lot complex biological processes. To obtain primary data about the PPI networks, the whole genome was uploaded in STRING Version 11.5 for the PPI network and functional enrichment analysis (Abadio et al., 2011; Hosen et al., 2014).

### 2.3 Interactome construction and analysis

Genes and gene connections with one another makes the PPI network. The tightly linked and most entangled node within a network is known as hub genes. The hub genes are determined through a maximal clique centrality (MCC) topological approach. To apply the MCC algorithm to the PPI network, CytoHubba was used. CytoHubba is basically a Cytoscape software plugin (http://apps.cytoscape.org/apps/cytohubba). The Cytoscape software plugin includes 11 topological algorithms for ranking nodes in a network (Hosen et al., 2014; Ali et al., 2020).

CYTOSCAPE version 2.8.2 was used for viewing the networks of protein already mentioned above. The NETWORK ANALYZER plug-in was used to analyze networks and the values of network centrality parameters such as closeness centrality (CC), betweenness centrality (BC), and degree centrality (DC). Gephi 0.8.2-beta was used to calculate the important measure of eigenvector centrality (EC). In order to categorize the top-ranked proteins/hubs in the network of important identified genes, the Java plug-in CytoHubba was used (Szklarczyk et al., 2019). Combined scores were taken from different parameters considered in STRING as edge weights for computing CytoHubba scores. Multiple topological algorithms such as Maximum Neighborhood Component (MNC), Maximal Clique Centrality (MCC), and Density of Maximum Neighborhood Component (DMNC) were used for finding important hub proteins in the AS and AST network.

### 2.4 Homology modeling and structure validation of the selected proteins

A structured model was created for both proteins guaA and metG through https://swissmodel.expasy.org/; i.e., protein sequences of both guaA and metG were submitted to https://swissmodel.expasy.org/ for structure prediction. Protein ID 7zu9.1.A was identified as a template for structure prediction for guaA and protein ID 6wq6.1.A was identified as a template for structure prediction for metG. Both the structures were validated through a Ramachandran plot (Arnold et al., 2006; Bordoli et al., 2009; Sobolev et al., 2020).

### 2.5 Binding site identification for virtual screening and docking

To prepare the selected protein for binding study, the first step was to identify its binding pockets using the CASTp server, which provides information about the protein’s surface topology. Once the binding pockets were identified, AutoDock v4.2 was used to create a grid box and map files for docking. The grid box coordinates, specifically *X*, *Y*, and *Z*, were saved in a grid parameter file (GPF) for future use. As the active site of protein is unknown, we followed a blind docking approach by enclosing the whole protein inside the grid (Ghersi and Sanchez, 2009). We used MGLTools v1.5.7 to execute GPF files and generate map files for docking purposes. After executing GPF files, we generated Grid log files (GLG) that contained all the atomic map files to be used as input parameters for docking. The program AU-TOGRID calculated the docking (Rizvi et al., 2013; Ramachandran et al., 2022).

### 2.6 Ligand screening

Virtual screening was used to identify small molecules that are most likely to bind to a target protein; ligand screening was performed with the help of Auto dock Vina (Li et al., 2019). Perl script was used to screen multiple ligands for selected proteins. Almost 100 different natural antimicrobial volatile organic compounds from multiple microbial and fungal isolates were selected for this study and are listed in **Supplementary Table 1**; they were screened to find out the best docked compounds. The 3D SDF structures for docking and screening purposes were obtained from PubChem (https://pubchem.ncbi.nlm.nih.gov/).

### 2.7 Protein ligand docking studies

Individual dockings were performed for guaA and metG against the top five ligands with high docking affinity and lower RMSD score. At the end of the docking process, the pose with the highest binding energy was chosen from the total conformations, and for each docking process, the binding orientations were analyzed. The docking studies were performed using Lamarckian Genetic Algorithm (LGA) (Morris et al., 2009). All the structural figures are generated using discovery studio 2021 (Pettersen et al., 2004). Virtual screening results were visually analyzed and the top ligand with a high docking affinity and a lower RMSD score was selected for docking purposes. Docking was performed with the help of AutoDock 4.2. The Genetic Algorithm parameter was selected for docking, while the minimum GA runs were set to be 50 and the population size was set to be 300 (Khattak et al., 2023). The number of evals was set to be 2,500,000 for 0 to 10 torsion angles, while it was set to be 250,000,000 for more than 10 torsion angles. The output was selected as Lamarckian GA.

### 2.8 Agar well diffusion assay

According to the methods of Elbeshehy et al. (2015), the antibacterial activity and inhibitory action of AgNPs were studied against the *A. oryzae* RS-1. *A. oryzae* RS-1 overnight culture of 200 μL (∼1 × 10^8^ CFU/mL) after being combined with 5 mL of half-strength LB medium was dispersed throughout an LB plate containing solid medium. The medium was air-dried first and then different doses of Enfumafungin (25, 50, and 100 μg/mL) were added evenly apart on the agar surface of the media plate and incubated at 30°C for 24 h. Finally, the diameter of the inhibition zone surrounding the wells was measured.

### 2.9 Minimum inhibitory concentration

A 96-well microtiter plate was used for the experiment of the minimum inhibitory concentration (MIC). In order to evaluate the MIC of Enfumafungin, 50 µL of bacterium cells from the *A. oryzae* RS-1 (1×10^8^ CFU/mL) was inoculated into Enfumafungin solution of different concentrations and various doses, with sterile DMSO serving as the control. Following that, the samples were incubated for 12 h at 30°C at 180 rpm. Using a Thermo Multiskan EX Microplate Photometer, the absorbance value at 600 nm was read to determine the quantity of bacteria present in the samples (Thermo Fisher Scientific Inc., Waltham, MA). This experiment was conducted three times with three replicates for each treatment.

### 2.10 Swarming and motility

LB plates containing 0.7% (w/v) agar were used to test the effect of Enfumafungin on *A. oryzae* RS-1 swarming motility. A semisolid LB agar plate containing 200 μg/mL Enfumafungin was loaded with 5 μL of overnight-cultured *A. oryzae* RS-1, while the plate devoid of Enfumafungin served as the control. The plates were then incubated at 30°C for 3 days. The colony diameter of strain RS-1 was assessed to quantify swarming motility, and the experiment was performed three times (Dong et al., 2016).

### 2.11 Live/dead cell staining

The live/dead staining approach was employed to determine whether the membrane of bacterial cells treated with Enfumafungin had been damaged or left undamaged according to the method developed by Ali et al. (2020), This was done by using the Invitrogen BacLight bacterial viability kit. Live bacteria were utilized as a positive control according to the kit’s protocol, and samples of dead bacteria that had been treated with isopropanol were used as a negative control. In order to treat bacterial cells independently as a positive control, negative control, and Enfumafungin treated, they were divided into three tubes, each of which contained 1 mL of bacterial cells. Fluorescence in the sample was detected using the Olympus inverted confocal microscope (Ali et al., 2020).

### 2.12 Biofilm inhibition assay

The efficacy of Enfumafungin to suppress the biofilm formation of Ao strain RS-1 was investigated using 96-well microtiter culture plates (Masum et al., 2018). *A. oryzae* strain RS-1 was cultured in LB broth until mid-exponential phase (∼1 × 10^8^ CFU/mL), and then 100 μL of bacterial culture with Enfumafungin at concentrations of 50 and 100 μg/mL were poured into each well. Sterile DMSO was applied as a control in place of Enfumafungin. For 24 h, the plates were held in static phase at 30°C, and after bacterial culture was disposed of, the 96-well plate was briefly rinsed with sterile ddH_2_O and dried for 24 h. The attached biofilm material was stained with 100 μL of 1% crystal violet (CV) solution and incubated at room temperature for 30–45 min. A SPECTRAmax®PLUS384 Microplate Spectrophotometer was then used to measure the absorbance at 570 nm of the leftover CV-stained solution after it had been solubilized with 200 μL of 33% acetic acid. The experiment was repeated three times while six replicates of each treatment were used.

### 2.13 Bacterial cell morphology observation using TEM

To prepare for TEM analysis, 1 mL of the Ao strain RS-1 bacterium (at approximately 1 × 10^8^ CFU/mL) was mixed with Enfumafungin to a final concentration of 50 μg/mL. The mixture served as the test sample, while the bacteria suspension without Enfumafungin served as the control. The bacteria were incubated in a shaker at 160 rpm and 30°C for 8 h. Afterwards, the bacterial cells were washed with 0.1 M PBS and fixed onto a glass slide with 2.5% glutaraldehyde. The samples were then dehydrated using a series of ethanol solutions at increasing concentrations (50%, 70%, 80%, 90%, 95%, and 100%) before being observed through TEM.

### 2.14 Statistical analysis

The experimental data were analyzed by IBM SPSS statistics version 23 for Windows software (SPSS Inc) and GraphPad Prism 7.00. The data represented show the average value with standard error of minimum three values of each independent experiment using one-way analysis of variance (ANOVA), Duncan’s test, and Student’s ***t*** *-*test to determine any significant differences between groups at *p* < 0.05.

## 3 Results and discussion

### 3.1 Genome analysis (Cytoscape network analysis)

Gene expression analysis utilizing microarray and RNA-seq datasets is considered to be sensitive for global gene expression investigations and finding probable molecular pathways that are activated in bacterial cells. We have studied and analyzed the genome analysis of bacterial strain *A. oryzae*, the causative agent of BBS. All the genes in the genome containing more than 2,500 individual genes were analyzed and the top 10 proteins of bacterial genome were selected for total protein analysis. These target proteins were selected on a range of basis, including closeness, entry, entry point, review status, protein type, and length of the proteins.

### 3.2 Features of the interactomes

A separate interactome of all bacterial protein was first constructed, which consists of all bacterial proteins and their interaction partners from the STRING (version 9.0) database. Furthermore, an integrated picture containing all the proteins and their connected partner proteins was taken into account to focus on the most indispensable proteins of the extremely complex phenotype. STRING is a comprehensive database that combines physical and functional interactions between proteins, utilizing various experimental and computational methods as well as public text collections. As it incorporates existing empirical and theoretical findings, it is possible that the network being analyzed may appear random (Morris et al., 1998) or exhibit a “small-world” topology, where each node has a similar number of connections (Watts and Strogatz, 1998). However, the interactomes in STRING follow a power law distribution without a characteristic scale, resulting in a scale-free network with a heavy tail degree distribution (Serafino et al., 2020). Taken together, the network and enrichment facilities in STRING enable comprehensive characterization of user gene lists and functional genomics datasets, and allow the creation and sharing of highly customized and augmented protein–protein association networks (Szklarczyk et al., 2021). PPIs play a crucial role in *in silico* drug discovery due to their significance in various biological processes and the potential they offer as therapeutic targets. PPIs can offer new opportunities for identifying novel drug targets. Inhibiting specific interactions can disrupt disease-related pathways, making them attractive candidates for drug discovery. Conventional drug targets (enzymes, receptors, etc.) have limitations. Targeting PPIs allows researchers to explore a broader range of therapeutic possibilities (Ran and Gestwicki, 2018). The whole data regarding the information about interactome and STRING database could not be provided because of the huge amount of data, but the data regarding the selected proteins’ name, accession numbers, locus, locus tag, and protein length are provided in **Table 1**.

**Table 1:** Data of selected proteins in the form of protein name, accession number, locus, locus tag, and protein length.

Accession	Protein name	Locus	Locus tag	Length
NZ_JMKU01000007.1	Glutamine-hydrolyzing GMP synthase	*guaA*	T336_RS0106180	541
NZ_JMKU01000001.1	Phosphoribosylformylglycinamidine synthase	*purL*	T336_RS0101125	1,340
NZ_JMKU01000017.1	Methionine-tRNA ligase	*metG*	T336_RS0113970	697
NZ_JMKU01000005.1	Triose-phosphate isomerase	*tpiA*	T336_RS0104230	251
NZ_JMKU01000032.1	30S ribosomal protein S3	*rpsC*	T336_RS0119710	293
NZ_JMKU01000001.1	Phosphoribosylamine-glycine ligase	*purD*	T336_RS0101715	426
NZ_KK366049.1	Isoleucine-tRNA ligase	*ileS*	T336_RS0122635	959
NZ_JMKU01000032.1	50S ribosomal protein L2	*rplB*	T336_RS0119695	274
NZ_JMKU01000007.1	Outer membrane protein assembly factor BamA	*bamA*	T336_RS0106695	765
NZ_JMKU01000002.1	MULTISPECIES: cell division protein FtsA	*ftsA*	T336_RS0102640	409
NZ_JMKU01000023.1	Membrane protein insertase YidC	*yidC*	T336_RS0117340	566
NZ_JMKU01000005.1	Lysine-tRNA ligase	*lysS*	T336_RS0104560	518
NZ_JMKU01000012.1	Histidinol dehydrogenase	*hisD*	T336_RS0111760	436
NZ_JMKU01000007.1	Translation initiation factor IF-2	*infB*	T336_RS0106260	946
NZ_JMKU01000032.1	MULTISPECIES: 50S ribosomal protein L4	*rplD*	T336_RS0119685	206
NZ_JMKU01000032.1	50S ribosomal protein L3	*rplC*	T336_RS0119680	224
NZ_JMKU01000010.1	MULTISPECIES: 30S ribosomal protein S7	*rpsG*	T336_RS0109670	157
NZ_JMKU01000032.1	MULTISPECIES: 50S ribosomal protein L22	*rplV*	T336_RS0119705	110

### 3.3 Top individual proteins and combined interactomes

**Figure 1:**
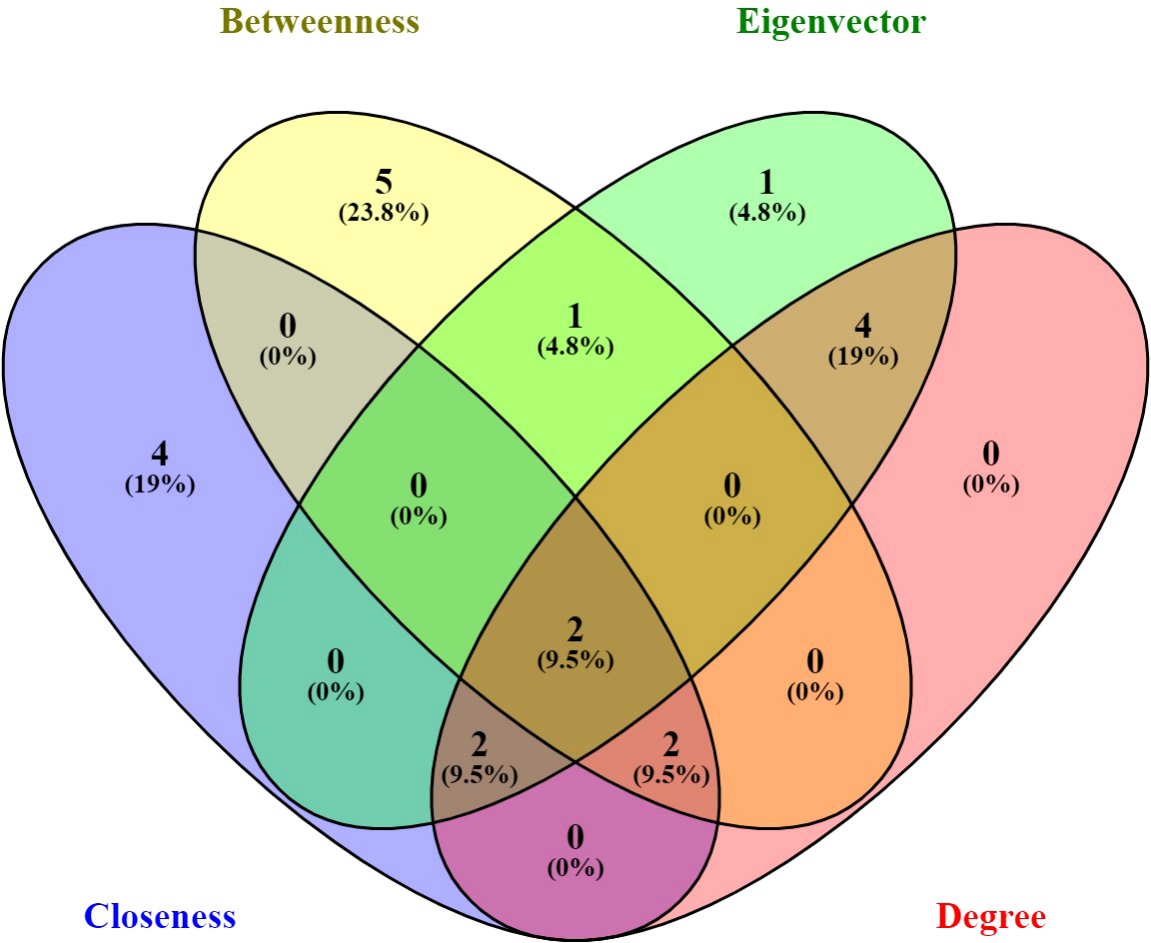
Venn diagram representation of all the genes present in each centrality; every centrality was shown in different color. Two genes common among betweenness centrality, eigenvector centrality, closeness centrality, and degree centrality. The image was created with the help of Venny 2.1.

The networks constructed in the study were analyzed using four important concepts in network analysis: DC, CC, BC, and EC. The focus was on identifying the proteins with the highest number of interacting partners, which were deemed most important. While DC is the most basic measure of centrality, it may not necessarily reflect the protein’s ability to carry out a specific function, such as attachment and invasion in the case of *A. oryzae*. CC, on the other hand, takes into account the protein’s ability to communicate sequentially with other proteins in the network, which could be more relevant to carrying out specific functions. Hub proteins have a high degree of connectivity, meaning they interact with many other proteins. These hubs often play crucial roles in various cellular functions and can be essential for processes like virulence in pathogens. Their central position in the network makes them potential targets for therapeutic intervention. BC can better reflect this fact than DC or CC since it measures the protein’s importance as a bridge between important hubs in the network. However, a protein’s ultimate importance lies in its connections to other important proteins in the network, which can be measured by EC. The top 10 important proteins were selected in each category on the basis of network analysis and values of BC, DC, CC, and EC. All the proteins were ranked in descending order based on their parameter values and consolidated in a table (**Supplementary Tables 3A, B**; **Figure 1**). The top 10 proteins selected in each category of BC, DC, CC, and EC were further analyzed with the help of Venny 2.1 (https://bioinfogp.cnb.csic.es/tools/venny/) to check for common proteins in the top 10 among BC, DC, CC, and EC.

### 3.4 Virtual screening and docking analysis

**Figure 2:**
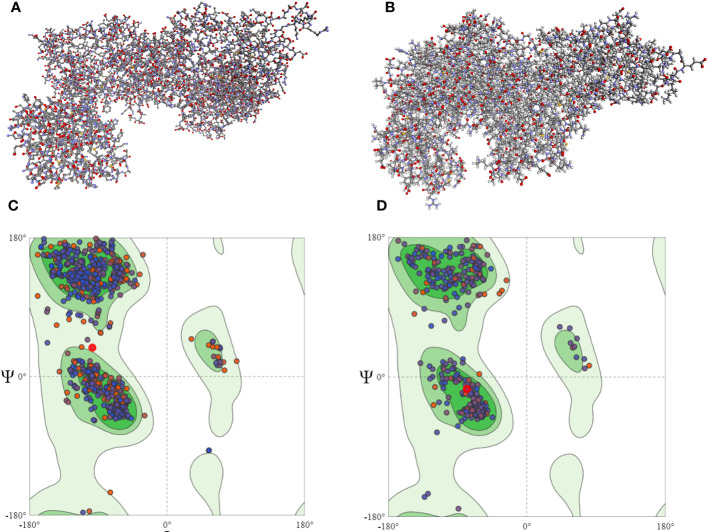
Homology modeled structure of guaA **(A)** and metG **(B)** proteins as well as Ramachandran plots for guaA **(C)** and metG **(D)**. The central green region represents the Ramachandran core region. Most of the protein residues in both panels **(C, D)** lie in the Ramachandran core region.

**Figure 3:**
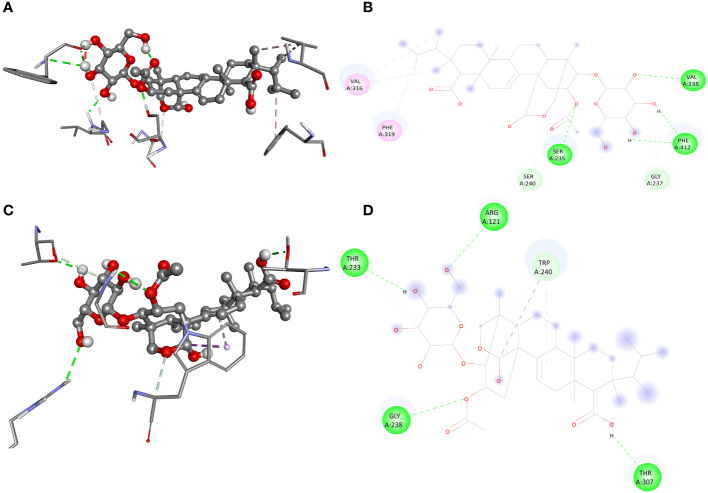
Interaction of enfumafungine with guaA shown in 3D **(A)**, 2D **(B)**, with metG in 3D **(C)** and 2D **(D)** format. The green lines represents hydrogen hydrogen bonding, the light green represents carbon hydrogen bonding, pink line represents alkyl or pi alkyl bonding while the purple lines represents pi sigma bonding.

The screening analysis of all the 100 selected compounds with the aforementioned proteins, viz., guaA and metG, which were already selected from the top ranker’s proteins on the basis of commonness in all centrality, was performed with help of Auto Dock Vina. All the selected drugs are listed in **Supplementary Table 1**. Before docking, a structured model was created for both proteins guaA and metG through https://swissmodel.expasy.org/. Both the structures were validated through a Ramachandran plot. For guaA, almost 96.19% of residues were in the favored region while only 0.28% was in the Ramachandran outlier region. For structure metG, almost 95.50% of residues were in the Ramachandran favored region while only 0.54% was in the Ramachandran outlier region (Bordoli et al., 2009). The structured model of both proteins guaA and metG and their Ramachandran plot are presented in **Figure 2**. The results of screening were analyzed in terms of scoring function of affinity and distance from best mode RMSD. The top five compounds having the lowest bonding energy in kcal/mol, high bonding affinity, and lowest RMSD were selected for docking studies. The data of ligand screening against the selected proteins are arranged in **Supplementary Tables 2A, B**. The docking of the top selected ligand among the 100 initial screened ligands was performed against the aforementioned proteins, viz., guaA and metG. The analysis of the docking results was based on the ranking of clusters of compound conformations with various ligands’ binding energy values. High affinity, indicating a strong binding between the ligand and protein, is associated with a high release of free energy during the binding process (Pantsar and Poso, 2018). Molecular docking is an important technique in structural molecular biology. The degree to which a single biomolecule binds to its ligand or another binding partner is referred to as its binding affinity (Seo et al., 2021). Among the top five compounds, Enfumafugin was selected for wet lab studies on the basis of the highest number of hydrogen bonds, lower binding energy, and lowest inhibition constant values against both guaA and metG. Based on the binding pattern of the aforementioned proteins, Enfumafugin upon binding with guaA showed a binding energy of −10.74, a reference RMSD value of 13.8, and an inhibition constant (KI) of 13.41 nM as shown in **Table 2**. Enfumafugin exhibits significant interactions in its binding with the protein. Notably, it engages in hydrogen–hydrogen (H–H) interactions with SER 235 and VAL 238, as well as two separate H–H interactions involving PHE 412. Additionally, there are two hydrogen–carbon (H–C) interactions, one with GLY 237 and another with SER 240. Furthermore, the compound forms three alkyl bond interactions, with two occurring at VAL 316 and one at PHE 319 of the guaA protein (as illustrated in **Figures 3A, B**). Regarding the binding characteristics, Enfumafugin demonstrates a binding energy of −7.81, accompanied by a reference RMSD value of 40.70 and an inhibition constant of 1.89 nM (as detailed in **Table 3**). Its interactions with amino acid residues within the protein are notably promising. Enfumafugin engages in H–H interactions with ARG 121, Thr 233, GLY 238, Trp 240, and Thr 307 (**Figures 3C, D**). A more favorable docking pose in the molecular docking analysis is suggested by a lower energy observed within the protein–ligand complex. This observation is consistent with the findings reported by Smith et al. (2015) and Schneider et al. (2011).

**Table 2:** Docking results of guaA protein with top five selected compounds in terms of reference RMSD values, binding energy values, inhibition constant, and number of hydrogen bonds.

No.	Protein	Compound	RMSD	Binding energy (kcal/mol)	Inhibition constant (KI)	Hydrogen bonds
1	guaA	B-sitosterol	12.7	−9.38	133.5 nM	2
2	guaA	Enfumafugin	13.8	−10.74	13.41 nM	5
3	guaA	Favolon	15	−10.59	17.37 nM	5
4	guaA	macrolactin_N	7.37	−9.41	125.6 nM	1
5	guaA	Neihumicin	15.0	−8.05	1.26 µM	2

**Table 3:** Docking results of metG protein with top five selected compounds in terms of reference RMSD values, binding energy values, inhibition constant, and number of hydrogen bonds.

No.	Protein	Compound	RMSD	Binding energy (kcal/mol)	Inhibition constant (KI)	Hydrogen bonds
1	metG	Enfumafugin	40.7	−7.81	1.89 nM	5
2	metG	Favolon	41.7	−8.28	845.7 nM	2
3	metG	Purpuromycin	29.5	−11.38	4.53 nM	4
4	metG	macrolactin_N	38.3	−8.37	733.6 nM	2
5	metG	Trypilepyrazinol	23.9	−8.04	1.28 µM	2

### 3.5 *In vitro* antibacterial activity of Enfumafungin

**Figure 4:**
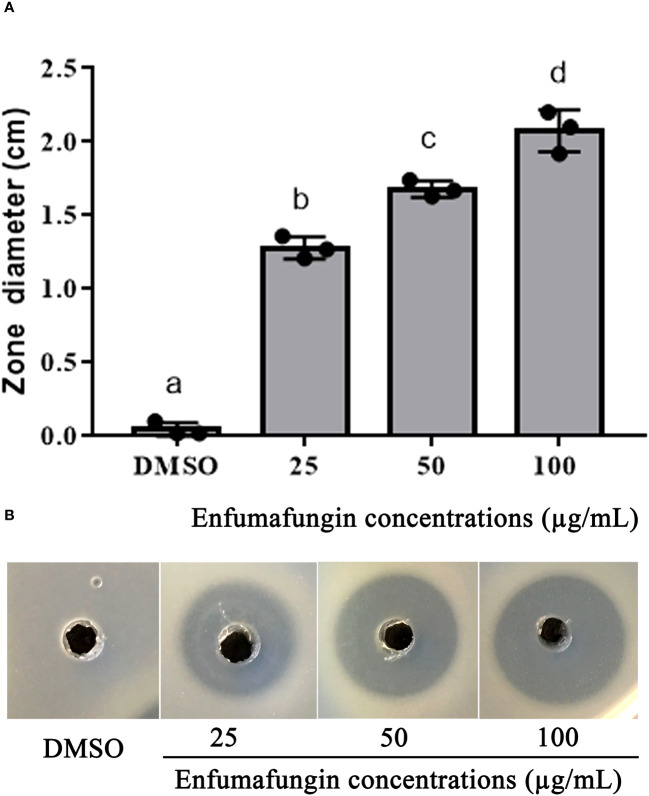
Enfumafungin inhibitory effect on *A. oryzae* RS-1. **(A)** The diameter of inhibition zones caused by different concentrations of Enfumafungin and compared to the control group consisting of DMSO solution. Error bars were included to account for the standard error (*n* = 3). Bars tagged with different letter(s) are statistically significant (*p* > 0.05). **(B)** The zone of inhibition achieved in agar well diffusion method produced by 25 µg/mL, 50 µg/mL, and 100 µg/mL Enfumafungin.

The agar well diffusion method is widely used to evaluate the antimicrobial activity of plants or microbial extracts (Magaldi et al., 2004; Valgas et al., 2007). Enfumafungin showed good antibacterial activity against *A. oryzae* strain RS-1 at three different concentrations after 24 h of incubation in the LB agar media. Increasing the concentration of Enfumafungin increased inhibition zone diameter. The highest zone was recorded to be 1.42 at 100 µg/mL (**Figure 4**). The second largest inhibition zone was achieved by a concentration of 50 µg/mL, followed by 25 µg/mL (**Figure 4**). Enfumafungin was originally thought to be an antifungal compound and proved to be very active against the *Candida* and *Aspergillus* species. It was also proved that Enfumafungin does not inhibit the growth of *Bacillus subtilis* (Onishi et al., 2000; Peláez et al., 2000). Overall results suggested that Enfumafungin showed a good selective antimicrobial activity against strain RS-1.

### 3.6 Minimum inhibitory concentration of Enfumafungin against *A. oryzae*

**Figure 5:**
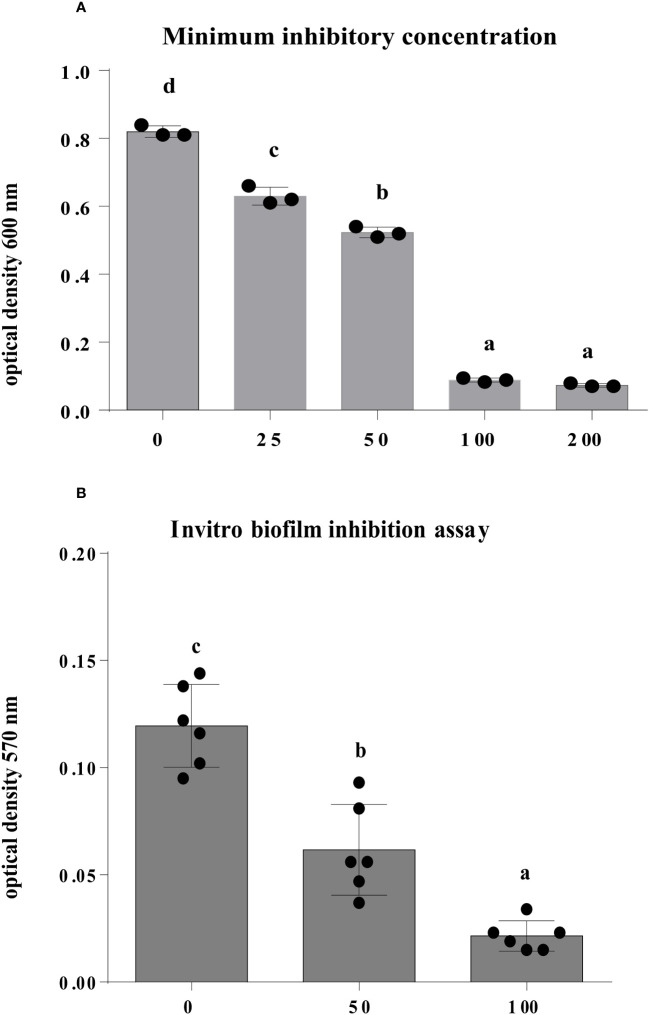
The antibacterial activity of Enfumafungin against *A. oryzae* RS-1. **(A)** The minimum inhibitory concentration and **(B)** biofilm inhibition assay of Enfumafungin against *A. oryzae* RS-1. The biofilm inhibition assay was after 30°C incubation without agitation for 24 h. Error bars were included to account for standard error (*n* = 3). Bars tagged with different letter(s) are statistically significant (*p* > 0.05).

The findings of this study showed that after 12 h of incubation, Enfumafungin has a discernible antibacterial activity against *A. oryzae* strain RS-1 in comparison to the control. Varying amounts of Enfumafungin had different antibacterial effects. Enfumafungin generally reduced the OD_600_ values by 23%, 36%, 54%, and 91% at doses of 25, 50, 100, and 200 µg/mL, respectively, while strain RS-1 showed the highest OD_600_ value (0.82) in the absence of Enfumafungin (**Figure 5A**). However, there was no noticeable difference in the antibacterial activity of Enfumafungin between the concentrations of 200 and 400 µg/mL, suggesting that Ao strain RS-1 is extremely susceptible to both Enfumafungin doses. The experimental MIC data strongly support the activity of Enfumafungin against *A. oryzae.* The hydrogen bonding is strongly correlated with the experimental values of MIC (Ramachandran et al., 2020).

### 3.7 Effect of Enfumafungin on biofilm formation of *A. oryzae*

We introduced a concentration of 50 and 100 µg/mL of Enfumafungin to strain RS-1 in a microtiter plate, and after 1 day of incubation at 30°C without stirring, the biofilm formation was substantially affected in comparison to control (**Figure 5B**). In fact, the *A. oryzae* strain RS-1 OD_570_ value was 0.1195 without Enfumafungin, compared to a lower OD_570_ value of 0.0625 and 0.0215 for strain RS-1 treated with 50 and 100 µg/mL Enfumafungin, respectively. Moreover, it was also found that Enfumafungin reduced biofilm of *A. oryzae* RS-1 in a dose-dependent manner as we treated *A. oryzae* RS-1 with 50 and 100 µg/mL. In general, the OD_570_ value of the *A. oryzae* strains RS-1 was reduced by 48% and 82%, respectively, as a result of the Enfumafungin compared to the control.

### 3.8 Enfumafungin affects swarming and motility of *A. oryzae*

**Figure 6:**
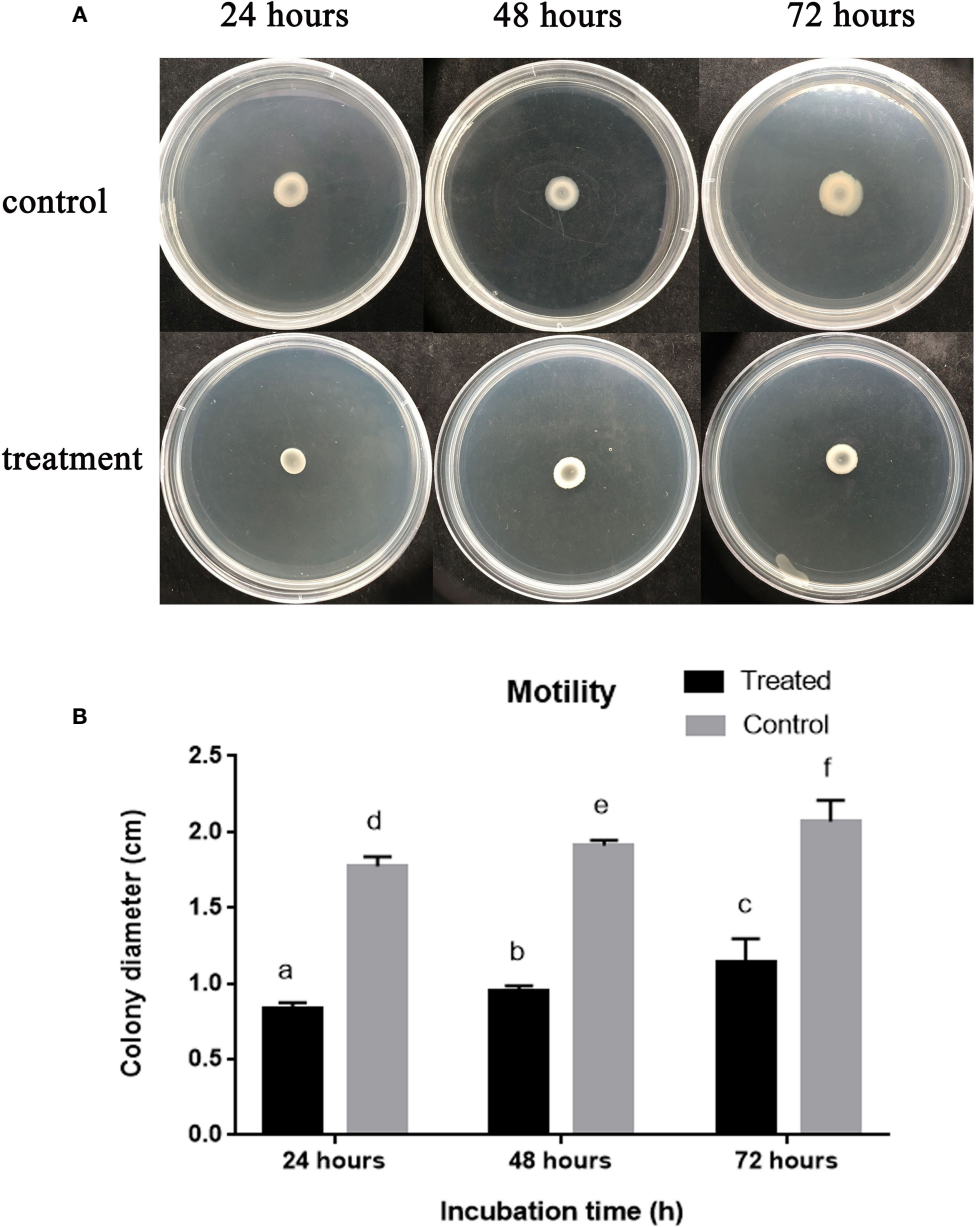
The effect of Enfumafungin on swarming motility of *A. oryzae* RS-1. **(A)** Showing bacterial colony in petri plate and **(B)** bacterial colony diameter after 24, 48, and 72 h. The concentration of Enfumafungin used for treated samples was 50 µg/mL. Error bars were included to account for standard error (*n* = 3). Bars tagged with different letter(s) are statistically significant (*p* > 0.05).

We carried out the *A. oryzae* strain RS-1 swarming motility assay to examine how AgNPs affect bacterial motility. The diameter of the bacterial colony was found to be 0.90, 1.14, and 1.30 cm after 24 h, 48 h, and 72 h, respectively, of incubation in the presence of 50 µg/mL Enfumafungin, as shown in **Figures 6A, B**, whereas the diameter of the bacterial colony was found to be 1.68, 1.81, and 1.9 after 24 h, 48 h, and 72 h of incubation, respectively. It is clear that Enfumafungin significantly inhibited the swarming motility of the *A. oryzae* strain RS-1 regardless of the incubation time. The swarming movement of *A. oryzae* strain RS-1 has been shown to be commonly related with bacterial growth, propagation, and pathogenicity (Masum et al., 2019); therefore, Enfumafungin inhibitory action is partially attributed to their ability to suppress this motility.

### 3.9 Bactericidal effect of Enfumafungin proved by live/dead cell staining

**Figure 7:**
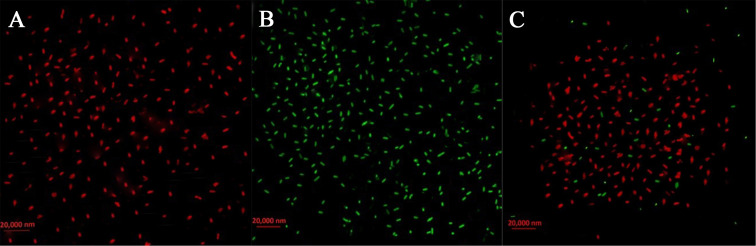
Live/dead cell staining of *A. oryzae* RS-1 treated with 100 µg/mL of Enfumafungin, using the BacLight bacterial viability kit and photographed by a fluorescence microscope. Live bacteria are shown by green fluorescence while dead bacteria are shown by red fluorescence. **(A)** Positive control showing live bacteria (without Enfumafungin). **(B)** Negative control showing dead bacteria treated with 95% ethanol treatment. **(C)** Bacteria treated with Enfumafungin.

Live/dead staining was utilized to see injured and healthy membranes in bacterial cells subjected to 200 µg/mL Enfumafungin in order to assess the viability of the bacteria. When a combination of red and green dyes was used to stain bacterial cells, only bacteria with intact cell membranes fluoresced green (**Figure 7**), whereas bacteria with injured cell membranes fluoresced red. It is interesting to note that, when exposed to Enfumafungin, bacterial cells displayed mostly red fluorescence. The results clearly shows that Enfumafungin has a bactericidal impact on the *A. oryzae* strain RS-1.

### 3.10 Enfumafungin damaged bacterial cell surface

**Figure 8:**
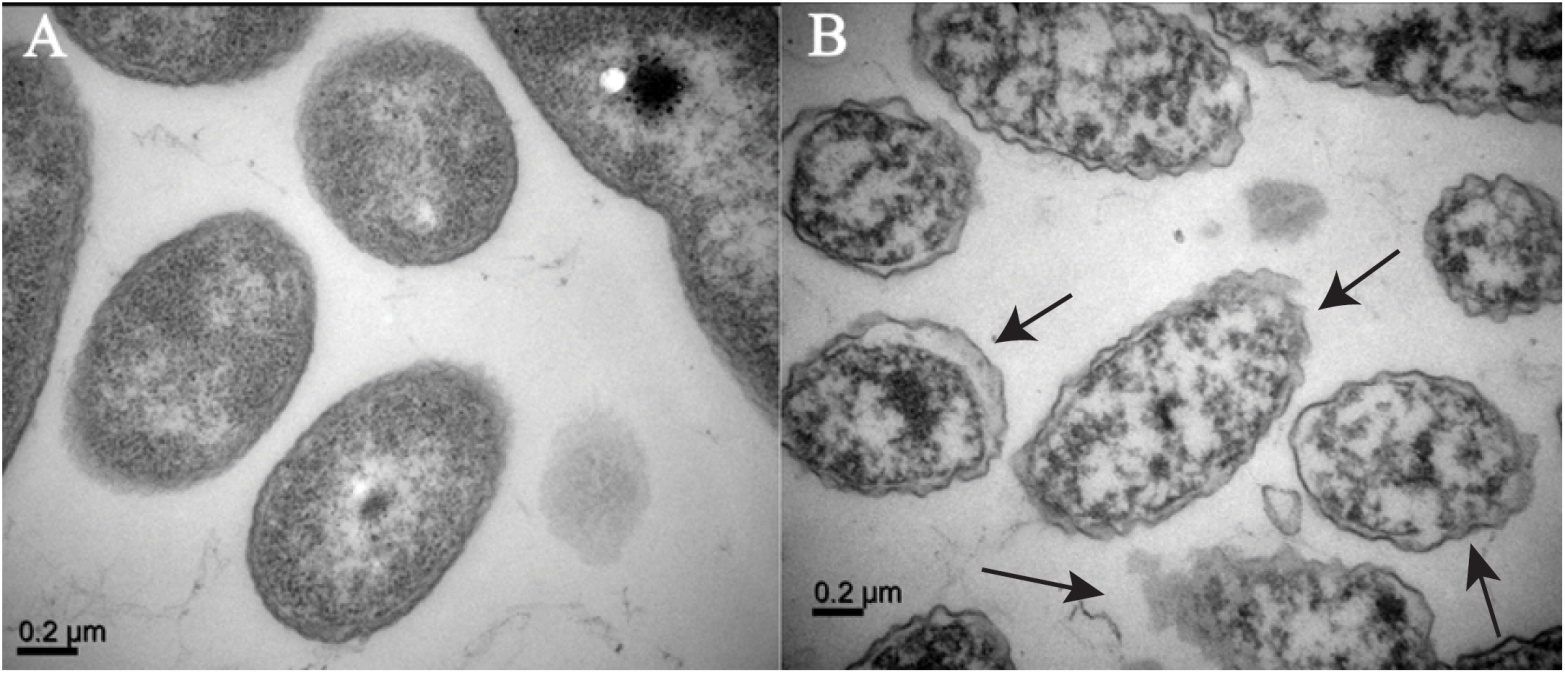
TEM observation of *A. oryzae* RS-1 treated without **(A)** and with **(B)** Enfumafungin 100 µg/mL. The arrows show broken and wrinkle cell membranes.

To assess the surface structure of the pretreated cells with Enfumafungin, TEM of the *A. oryzae* strain RS-1 was performed. **Figure 8A** shows bacterial cell morphology prior to treatment, which are mostly round-shaped bacterial forms. **Figure 8B** shows the extreme abnormalities of the cells following treatment with Enfumafungin. **Figure 8B** shows that when the cells were treated with Enfumafungin, the cell wall and membrane became curved and damaged, but the untreated bacterial cell wall and membrane remained undamaged. TEM images revealed both severe and extensive cell wall destruction, which led to protein and nucleic acid leaks and ultimately led to bacterial mortality (Ali et al., 2020).

Enfumafungin is an expensive antifungal agent, but large-scale production of Enfumafungin on a commercial basis will decrease its price and will become affordable. As production volumes increase, the per-unit cost tends to decrease due to economies of scale (Stigler, 1958). Apart from that automation, reducing waste, improving energy efficiency, and enhancing the overall production workflow will also decrease the price (Christiaanse and Hulstijn, 2012). So far, there is no study or report of toxicity of Enfumafungin against humans or animals; in the future, if it happens, there are several measures we can take to reduce the amount of toxicity caused by Enfumafungin. We can use Enfumafungin as a seed priming agent instead of spraying it directly on a rice field. With multiple field trials, we can assess the amount of Enfumafungin accumulated in rice grains, and then we can easily assess the amount of toxicity caused by Enfumafungin; hence, we can optimize dose to avoid toxicity. As every chemical is degradable and has a specific half-life at a specific temperature and in a specific environment, with multiple field trials, we can assess the half-life of Enfumafungin in different conditions and can easily avoid Enfumafungin toxicity.

## 4 Conclusion

Multidrug resistance has increased dramatically in the last two decades. Therefore, drug repurposing is gaining importance. The analysis and prediction of the activity of existing and novel drug ligands for new protein targets are based on the concept that similar compounds tend to have similar biological properties. Similarly, incorporating a structure-based approach focuses on obtaining proteins likely to have similar functions and/or to recognize similar ligands. Thus, in the field of drug repurposing, protein comparison is used as a method to identify secondary targets of an approved drug. To hypothesize a new target for treating BBS disease in *O. sativa*, we have explored the results of a network-based approach to identify the most indispensable proteins important for next-generation drugs (Peláez et al., 2000). The present study further develops this work at a structural level. From our study, proteins guaA and metG have been demonstrated to be potential druggable targets for the new ligand Enfumafugin. Thus, Enfumafugin confers high binding affinity toward the target proteins guaA and metG with a binding affinity comparable to the approved drugs. Enfumafugin is a strong antifungal compound while our study suggests that Enfumafugin is a selective antibacterial compound that inhibits *A. oryzae* growth at a concentration of 100 µg/mL, which a major breakthrough in the field of drug discovery.

## Data availability statement

The datasets presented in this study can be found in online repositories. The names of the repository/repositories and accession number(s) can be found in the article/**Supplementary Material**.

## Author contributions

Conceptualization, AH and AT; methodology, AA; software, AK and JQ; validation, EI, AK, and TA; formal analysis, ZK; investigation, AT, JQ, AH, MA-D, and HA; resources, AT and LX; data curation, AT; writing—original draft preparation, AT; writing—review and editing, AH, MA-D, HA, and ZK; visualization, ZK; supervision, BL; project administration, BL; funding acquisition, LX, HA, and BL. All authors contributed to the article and approved the submitted version.

## References

[B1] Abadio A. K. R. Kioshima E. S. Teixeira M. M. Martins N. F. Maigret B. Felipe M. S. S. (2011). Comparative genomics allowed the identification of drug targets against human fungal pathogens. BMC Genomics 12, 1–10. doi: 10.1186/1471-2164-12-75 PMC3042012 21272313

[B2] Ali K. A. Yao R. Wu W. Masum M. M. I. Luo J. Wang Y.. (2020). Biosynthesis of silver nanoparticle from pomelo (Citrus Maxima) and their antibacterial activity against acidovorax oryzae RS-2. Mater. Res. Express 7, 015097. doi: 10.1088/2053-1591/ab6c5e

[B3] An J.-X. Ma Y. Zhao W.-B. Hu Y.-M. Wang Y.-R. Zhang Z.-J.. (2023). Drug repurposing strategy II: From approved drugs to agri-fungicide leads. J. Antibiotics 76, 131–182. doi: 10.1038/s41429-023-00594-2 PMC9880955 36707717

[B4] Arnold K. Bordoli L. Kopp J. Schwede T. (2006). The SWISS-MODEL workspace: a web-based environment for protein structure homology modelling. Bioinformatics 22, 195–201. doi: 10.1093/bioinformatics/bti770 16301204

[B5] Aubé J. (2012). Drug repurposing and the medicinal chemist. ACS medicinal chemistry letters 3(6), 442–444. 10.1021/ml300114c PMC4025634 24900492

[B6] Bordoli L. Kiefer F. Arnold K. Benkert P. Battey J. Schwede T. (2009). Protein structure homology modeling using SWISS-MODEL workspace. Nat. Protoc. 4, 1–13. doi: 10.1038/nprot.2008.197 19131951

[B7] Christiaanse R. Hulstijn J. (2012). “Control automation to reduce costs of control,” in Advanced Information Systems Engineering Workshops: CAiSE 2012 International Workshops, Gdańsk, Poland, June 25-26, 2012. Proceedings 24. 322–336 (Springer).

[B8] Csermely P. Korcsmáros T. Kiss H. J. London G. Nussinov R. (2013). Structure and dynamics of molecular networks: a novel paradigm of drug discovery: a comprehensive review. Pharmacol. Ther. 138, 333–408. doi: 10.1016/j.pharmthera.2013.01.016 23384594 PMC3647006

[B9] Dai Y.-F. Zhao X.-M. (2015). A survey on the computational approaches to identify drug targets in the postgenomic era. BioMed. Res. Int. 9. doi: 10.1155/2015/239654 PMC4427773 26060814

[B10] Dong Q. Luo J. Qiu W. Cai L. Anjum S. I. Li B.. (2016). Inhibitory effect of camptothecin against rice bacterial brown stripe pathogen Acidovorax avenae subsp. avenae RS-2. Molecules 21, 978. doi: 10.3390/molecules21080978 27472315 PMC6274382

[B11] Elbeshehy E. K. Elazzazy A. M. Aggelis G. (2015). Silver nanoparticles synthesis mediated by new isolates of Bacillus spp., nanoparticle characterization and their activity against Bean Yellow Mosaic Virus and human pathogens. Front. Microbiol. 6, 453. doi: 10.3389/fmicb.2015.00453 26029190 PMC4429621

[B12] Ghersi D. Sanchez R. (2009). Improving accuracy and efficiency of blind protein-ligand docking by focusing on predicted binding sites. Proteins: Struct. Funct. Bioinf. 74, 417–424. doi: 10.1002/prot.22154 PMC2610246 18636505

[B13] Hosen M. I. Tanmoy A. M. Mahbuba D.-A. Salma U. Nazim M. Islam M. T.. (2014). Application of a subtractive genomics approach for in silico identification and characterization of novel drug targets in Mycobacterium tuberculosis F11. Interdiscip. Sci.: Comput. Life Sci. 6, 48–56. doi: 10.1007/s12539-014-0188-y 24464704

[B14] Katsila T. Spyroulias G. A. Patrinos G. P. Matsoukas M.-T. (2016). Computational approaches in target identification and drug discovery. Comput. Struct. Biotechnol. J. 14, 177–184. doi: 10.1016/j.csbj.2016.04.004 27293534 PMC4887558

[B15] Khattak A. A. Ahmad A. Khattak H. A. Mz I. K. (2023). Identification of small inhibitors for human Metadherin, an oncoprotein, through Insilco approach. Curr. Computer-aided Drug Design 19(4), 278–287. doi: 10.2174/1573409919666230110112356 36627784

[B16] Laxminarayan R. Duse A. Wattal C. Zaidi A. K. Wertheim H. F. Sumpradit N.. (2013). Antibiotic resistance—the need for global solutions. Lancet Infect. Dis. 13, 1057–1098. doi: 10.1016/S1473-3099(13)70318-9 24252483

[B18] Li X. Liu Z. Mi M. Zhang C. Xiao Y. Liu X.. (2019). Identification of hub genes and key pathways associated with angioimmunoblastic T-cell lymphoma using weighted gene co-expression network analysis. Cancer Manage. Res., 5209–5220. doi: 10.2147/CMAR.S185030 PMC6559227 31239775

[B17] Li B. Wang L. Ibrahim M. Ge M. Wang Y. Mannan S.. (2015). Membrane protein profiling of Acidovorax avenae subsp. avenae under various growth conditions. Arch. Microbiol. 197, 673–682. doi: 10.1007/s00203-015-1100-9 25763989

[B19] Magaldi S. Mata-Essayag S. De Capriles C. H. Pérez C. Colella M. Olaizola C.. (2004). Well diffusion for antifungal susceptibility testing. Int. J. Infect. Dis. 8, 39–45. doi: 10.1016/j.ijid.2003.03.002 14690779

[B20] March-Vila E. Pinzi L. Sturm N. Tinivella A. Engkvist O. Chen H.. (2017). On the integration of in silico drug design methods for drug repurposing. Front. Pharmacol. 298. doi: 10.3389/fphar.2017.00298 PMC5440551 28588497

[B1000] Masum M. Liu L. Yang M. Hossain M. Siddiqa M. Supty M. (2017). Halotolerant bacteria belonging to operational group Bacillus amyloliquefaciens in biocontrol of the rice brown stripe pathogen Acidovorax oryzae. Journal of applied microbiology 125, 1852-1867. 10.1111/jam.14088 30146698

[B21] Masum M. M. I. Siddiqa M. M. Ali K. A. Zhang Y. Abdallah Y. Ibrahim E.. (2019). Biogenic synthesis of silver nanoparticles using Phyllanthus emblica fruit extract and its inhibitory action against the pathogen Acidovorax oryzae strain RS-2 of rice bacterial brown stripe. Front. Microbiol. 10, 820. doi: 10.3389/fmicb.2019.00820 31110495 PMC6501729

[B22] Morris G. M. Goodsell D. S. Halliday R. S. Huey R. Hart W. E. Belew R. K.. (1998). Automated docking using a Lamarckian genetic algorithm and an empirical binding free energy function. J. Comput. Chem. 19, 1639–1662. doi: 10.1002/(SICI)1096-987X(19981115)19:14<1639::AID-JCC10>3.0.CO;2-B

[B23] Morris G. M. Huey R. Lindstrom W. Sanner M. F. Belew R. K. Goodsell D. S.. (2009). AutoDock4 and AutoDockTools4: Automated docking with selective receptor flexibility. J. Comput. Chem. 30, 2785–2791. doi: 10.1002/jcc.21256 19399780 PMC2760638

[B24] Onishi J. Meinz M. Thompson J. Curotto J. Dreikorn S. Rosenbach M.. (2000). Discovery of novel antifungal (1, 3)-β-D-glucan synthase inhibitors. Antimicrob. Agents Chemother. 44, 368–377. doi: 10.1128/AAC.44.2.368-377.2000 10639364 PMC89685

[B25] Pan A. Lahiri C. Rajendiran A. Shanmugham B. (2016). Computational analysis of protein interaction networks for infectious diseases. Briefings Bioinf. 17, 517–526. doi: 10.1093/bib/bbv059 PMC7110031 26261187

[B26] Pantsar T. Poso A. (2018). Binding affinity *via* docking: fact and fiction. Molecules 23, 1899. doi: 10.3390/molecules23081899 30061498 PMC6222344

[B27] Peláez F. Cabello A. Platas G. Díez M. T. Del Val A. G. Basilio A.. (2000). The discovery of enfumafungin, a novel antifungal compound produced by an endophytic Hormonema species biological activity and taxonomy of the producing organisms. System. Appl. Microbiol. 23, 333–343. doi: 10.1016/S0723-2020(00)80062-4 11108011

[B28] Pettersen E. F. Goddard T. D. Huang C. C. Couch G. S. Greenblatt D. M. Meng E. C.. (2004). UCSF Chimera—a visualization system for exploratory research and analysis. J. Comput. Chem. 25, 1605–1612. doi: 10.1002/jcc.20084 15264254

[B29] Ramachandran B. Jeyakanthan J. Lopes B. S. (2020). Molecular docking, dynamics and free energy analyses of Acinetobacter baumannii OXA class enzymes with carbapenems investigating their hydrolytic mechanisms. J. Med. Microbiol. 69, 1062–1078. doi: 10.1099/jmm.0.001233 32773005

[B30] Ramachandran B. Srinivasadesikan V. Chou T.-M. Jeyakanthan J. Lee S.-L. (2022). Atomistic simulation on flavonoids derivatives as potential inhibitors of bacterial gyrase of Staphylococcus aureus. J. Biomol. Struct. Dyn. 40, 4314–4327. doi: 10.1080/07391102.2020.1856184 33308046

[B31] Ran X. Gestwicki J. E. (2018). Inhibitors of protein–protein interactions (PPIs): an analysis of scaffold choices and buried surface area. Curr. Opin. Chem. Biol. 44, 75–86. doi: 10.1016/j.cbpa.2018.06.004 29908451 PMC6066447

[B32] Rangel-Vega A. Bernstein L. R. Mandujano-Tinoco E. A. García-Contreras S. J. García-Contreras R. (2015). Drug repurposing as an alternative for the treatment of recalcitrant bacterial infections. Front. Microbiol. 6, 282. doi: 10.3389/fmicb.2015.00282 25914685 PMC4391038

[B33] Rizvi S. M. D. Shakil S. Haneef M. (2013). A simple click by click protocol to perform docking: AutoDock 4.2 made easy for non-bioinformaticians. EXCLI J. 12, 831. 26648810 PMC4669947

[B34] Schneider K. D. Ortega C. J. Renck N. A. Bonomo R. A. Powers R. A. Leonard D. A. (2011). Structures of the class D carbapenemase OXA-24 from Acinetobacter baumannii in complex with doripenem. J. Mol. Biol. 406, 583–594. doi: 10.1016/j.jmb.2010.12.042 21215758 PMC3057435

[B35] Sekyere J. O. Asante J. (2018). Emerging mechanisms of antimicrobial resistance in bacteria and fungi: advances in the era of genomics. Future Microbiol. 13, 241–262. doi: 10.2217/fmb-2017-0172 29319341

[B36] Seo S. Choi J. Park S. Ahn J. (2021). Binding affinity prediction for protein–ligand complex using deep attention mechanism based on intermolecular interactions. BMC Bioinf. 22, 1–15. doi: 10.1186/s12859-021-04466-0 PMC8576937 34749664

[B37] Serafino M. Cimini G. Maritan A. Rinaldo A. Suweis S. Banavar J. R.. (2020). True scale-free networks hidden by finite size effects. Phys. Sci. 118. doi: 10.1073/pnas.2013825118 PMC7812829 33380456

[B38] Shanmugam G. Jeon J. (2017). Computer-aided drug discovery in plant pathology. Plant Pathol. J. 33, 529. doi: 10.5423/PPJ.RW.04.2017.0084 29238276 PMC5720600

[B39] Sheikh H. M. A. (2010). Antimicrobial activity of certain bacteria and fungi isolated from soil mixed with human saliva against pathogenic microbes causing dermatological diseases. Saudi J. Biol. Sci. 17, 331–339. doi: 10.1016/j.sjbs.2010.06.003 30323712 PMC6181150

[B40] Smith C. A. Antunes N. T. Stewart N. K. Frase H. Toth M. Kantardjieff K. A.. (2015). Structural basis for enhancement of carbapenemase activity in the OXA-51 family of class D β-lactamases. ACS Chem. Biol. 10, 1791–1796. doi: 10.1021/acschembio.5b00090 26042471 PMC4546549

[B41] Sobolev O. V. Afonine P. V. Moriarty N. W. Hekkelman M. L. Joosten R. P. Perrakis A.. (2020). A global Ramachandran score identifies protein structures with unlikely stereochemistry. Structure 28, 1249–1258. doi: 10.1016/j.str.2020.08.005 32857966 PMC7642142

[B42] Song W. Kim H. Hwang C. Schaad N. (2004). Detection of Acidovorax avenae ssp. avenae in rice seeds using BIO-PCR. J. Phytopathol. 152, 667–676. doi: 10.1111/j.1439-0434.2004.00914.x

[B43] Stigler G. J. (1958). The economies of scale. J. Law Econ. 1, 54–71. doi: 10.1086/466541

[B44] Szklarczyk D. Gable A. L. Lyon D. Junge A. Wyder S. Huerta-Cepas J.. (2019). STRING v11: protein–protein association networks with increased coverage, supporting functional discovery in genome-wide experimental datasets. Nucleic Acids Res. 47, D607–D613. doi: 10.1093/nar/gky1131 30476243 PMC6323986

[B45] Szklarczyk D. Gable A. L. Nastou K. C. Lyon D. Kirsch R. Pyysalo S.. (2021). The STRING database in 2021: customizable protein–protein networks, and functional characterization of user-uploaded gene/measurement sets. Nucleic Acids Res. 49, D605–D612. doi: 10.1093/nar/gkab835 33237311 PMC7779004

[B46] Szklarczyk D. Morris J. H. Cook H. Kuhn M. Wyder S. Simonovic M.. (2016). The STRING database in 2017: quality-controlled protein–protein association networks, made broadly accessible. Nucleic Acids Res. 45, gkw937. doi: 10.1093/nar/gkw937 PMC5210637 27924014

[B47] Valgas C. Souza S. M. D. Smânia E. F. Smânia,. A. Jr (2007). Screening methods to determine antibacterial activity of natural products. Braz. J. Microbiol. 38, 369–380. doi: 10.1590/S1517-83822007000200034

[B48] Watts D. J. Strogatz S. H. (1998). Collective dynamics of ‘small-world’networks. nature 393, 440–442. doi: 10.1038/30918 9623998

[B49] Wooller S. K. Benstead-Hume G. Chen X. Ali Y. Pearl F. M. (2017). Bioinformatics in translational drug discovery. Biosci. Rep. 37. doi: 10.1042/BSR20160180 PMC6448364 28487472

[B50] Xie G.-L. Zhang G.-Q. Liu H. Lou M.-M. Tian W.-X. Li B.. (2011). Genome sequence of the rice-pathogenic bacterium Acidovorax avenae subsp. avenae RS-1. Am. Soc. Microbiol. 5013–5014. 10.1128/JB.05594-11 PMC3165674 21742879

