# Supplementary material for: Precision drug design against Acidovorax oryzae: leveraging bioinformatics to combat rice brown stripe disease
Source: Front Cell Infect Microbiol. 2023 Oct 11;13:1225285. doi: 10.3389/fcimb.2023.1225285 (PMC10598866; doi:10.3389/fcimb.2023.1225285)
Supplement: Supplementary file 1 [file Table_1.docx]

**Table S1.** Selected drugs for screening against protein guaA and metG along with their references.

| S.No | Compound name | Reference |
| --- | --- | --- |
| 1 | Enfumafungin | Pelaez, F.; Cabello, A.; Platas, G.; Díez, M.T.; Del Val, A.G.; Basilio, A.; Martán, I.; Vicente, F.; Bills, G.F.; Giacobbe, R.A.; et al. The discovery of enfumafungin, a novel antifungal compound produced by an endophytic Hormonema species biological activity and taxonomy of the producing organisms. Syst. Appl. Microbiol. **2000**, 23, 333–343. |
| 2 | Favolon | Chepkirui, C.; Richter, C.; Matasyoh, J.C.; Stadler, M. Monochlorinated calocerins A–D and 9-oxostrobilurin derivatives from the  basidiomycete Favolaschia calocera. Phytochemistry **2016**, 132, 95–101. |
| 3 | Rubrolide S | Zhu, T.; Chen, Z.; Liu, P.; Wang, Y.; Xin, Z.; Zhu, W. New rubrolides from the marine-derived fungus Aspergillus terreus  OUCMDZ-1925. J. Antibiot. **2014**, 67, 315–318. |
| 4 | Nisin A | Carroll, J.; Draper, L.A.; O’Connor, P.M.; Coffey, A.; Hill, C.; Ross, R.P.; Cotter, P.D.; O’Mahony, J. Comparison of the activities  of the lantibiotics nisin and lacticin 3147 against clinically significant mycobacteria. Int. J. Antimicrob. Agents **2010**, 36, 132–136. |
| 5 | Pradimicins A, B, C | Tomita, K.; Nishio, M.; Saitoh, K.; Yamamoto, H.; Hoshino, Y.; Ohkuma, H.; Konishi, M.; Miyaki, T.; Oki, T. Pradimicins A, B and C: New antifungal antibiotics. I. Taxonomy, production, isolation and physico-chemical properties. J. Antibiot. **1990**, 43, 755–762. |
| 6 | Purpuromycin | Kerr, J.R. Bacterial inhibition of fungal growth and pathogenicity. Microb. Ecol. Health Dis. **1999**, 11, 129–142. |
| 7 | Neihumicin | Boumehira, A.Z.; El-Enshasy, H.A.; Hacene, H.; Elsayed, E.A.; Aziz, R.; Park, E.Y. Recent progress on the development of  antibiotics from the genus Micromonospora. Biotechnol. Bioprocess Eng. **2016**, 21, 199–223. |
| 8 | Azoxybacilin | Kerr, J.R. Bacterial inhibition of fungal growth and pathogenicity. Microb. Ecol. Health Dis. **1999**, 11, 129–142. |
| 9 | Cispentacin | Kerr, J.R. Bacterial inhibition of fungal growth and pathogenicity. Microb. Ecol. Health Dis. **1999**, 11, 129–142. |
| 10 | Sch 37137 | Schwartz, R.E.; Giacobbe, R.A.; Monaghan, R.L. L-671,329, a new antifungal agent. I. Fermentation and isolation. J. Antibiot. **1989**, 42, 163–167. |
| 11 | 2-Butanone | Garrido, A., Atencio, L.A., Bethancourt, R., Bethancourt, A., Guzmán, H., Gutiérrez, M. and Durant-Archibold, A.A., 2020. Antibacterial Activity of Volatile Organic Compounds Produced by the Octocoral-Associated Bacteria Bacillus sp. BO53 and Pseudoalteromonas sp. GA327. *Antibiotics*, *9*(12), p.923. |
| 12 | 1-Butanol | Garrido, A., Atencio, L.A., Bethancourt, R., Bethancourt, A., Guzmán, H., Gutiérrez, M. and Durant-Archibold, A.A., 2020. Antibacterial Activity of Volatile Organic Compounds Produced by the Octocoral-Associated Bacteria Bacillus sp. BO53 and Pseudoalteromonas sp. GA327. *Antibiotics*, *9*(12), p.923. |
| 13 | 2,2,4-Trimethylpentane | Garrido, A., Atencio, L.A., Bethancourt, R., Bethancourt, A., Guzmán, H., Gutiérrez, M. and Durant-Archibold, A.A., 2020. Antibacterial Activity of Volatile Organic Compounds Produced by the Octocoral-Associated Bacteria Bacillus sp. BO53 and Pseudoalteromonas sp. GA327. *Antibiotics*, *9*(12), p.923. |
| 14 | 2-Pentanone | Garrido, A., Atencio, L.A., Bethancourt, R., Bethancourt, A., Guzmán, H., Gutiérrez, M. and Durant-Archibold, A.A., 2020. Antibacterial Activity of Volatile Organic Compounds Produced by the Octocoral-Associated Bacteria Bacillus sp. BO53 and Pseudoalteromonas sp. GA327. *Antibiotics*, *9*(12), p.923. |
| 15 | 1-Pentanol | Garrido, A., Atencio, L.A., Bethancourt, R., Bethancourt, A., Guzmán, H., Gutiérrez, M. and Durant-Archibold, A.A., 2020. Antibacterial Activity of Volatile Organic Compounds Produced by the Octocoral-Associated Bacteria Bacillus sp. BO53 and Pseudoalteromonas sp. GA327. *Antibiotics*, *9*(12), p.923. |
| 16 | Butyl formate | Garrido, A., Atencio, L.A., Bethancourt, R., Bethancourt, A., Guzmán, H., Gutiérrez, M. and Durant-Archibold, A.A., 2020. Antibacterial Activity of Volatile Organic Compounds Produced by the Octocoral-Associated Bacteria Bacillus sp. BO53 and Pseudoalteromonas sp. GA327. *Antibiotics*, *9*(12), p.923. |
| 17 | o-Xylene | Garrido, A., Atencio, L.A., Bethancourt, R., Bethancourt, A., Guzmán, H., Gutiérrez, M. and Durant-Archibold, A.A., 2020. Antibacterial Activity of Volatile Organic Compounds Produced by the Octocoral-Associated Bacteria Bacillus sp. BO53 and Pseudoalteromonas sp. GA327. *Antibiotics*, *9*(12), p.923. |
| 18 | 6-Methyl-5-heptene-2-one | Garrido, A., Atencio, L.A., Bethancourt, R., Bethancourt, A., Guzmán, H., Gutiérrez, M. and Durant-Archibold, A.A., 2020. Antibacterial Activity of Volatile Organic Compounds Produced by the Octocoral-Associated Bacteria Bacillus sp. BO53 and Pseudoalteromonas sp. GA327. *Antibiotics*, *9*(12), p.923. |
| 19 | p-Cymene | Garrido, A., Atencio, L.A., Bethancourt, R., Bethancourt, A., Guzmán, H., Gutiérrez, M. and Durant-Archibold, A.A., 2020. Antibacterial Activity of Volatile Organic Compounds Produced by the Octocoral-Associated Bacteria Bacillus sp. BO53 and Pseudoalteromonas sp. GA327. *Antibiotics*, *9*(12), p.923. |
| 20 | Benzyl Alcohol | Garrido, A., Atencio, L.A., Bethancourt, R., Bethancourt, A., Guzmán, H., Gutiérrez, M. and Durant-Archibold, A.A., 2020. Antibacterial Activity of Volatile Organic Compounds Produced by the Octocoral-Associated Bacteria Bacillus sp. BO53 and Pseudoalteromonas sp. GA327. *Antibiotics*, *9*(12), p.923. |
| 21 | 3-Methylacetophenone | Garrido, A., Atencio, L.A., Bethancourt, R., Bethancourt, A., Guzmán, H., Gutiérrez, M. and Durant-Archibold, A.A., 2020. Antibacterial Activity of Volatile Organic Compounds Produced by the Octocoral-Associated Bacteria Bacillus sp. BO53 and Pseudoalteromonas sp. GA327. *Antibiotics*, *9*(12), p.923. |
| 22 | 2-Undecanone | Garrido, A., Atencio, L.A., Bethancourt, R., Bethancourt, A., Guzmán, H., Gutiérrez, M. and Durant-Archibold, A.A., 2020. Antibacterial Activity of Volatile Organic Compounds Produced by the Octocoral-Associated Bacteria Bacillus sp. BO53 and Pseudoalteromonas sp. GA327. *Antibiotics*, *9*(12), p.923. |
| 23 | 2-Heptanone | Garrido, A., Atencio, L.A., Bethancourt, R., Bethancourt, A., Guzmán, H., Gutiérrez, M. and Durant-Archibold, A.A., 2020. Antibacterial Activity of Volatile Organic Compounds Produced by the Octocoral-Associated Bacteria Bacillus sp. BO53 and Pseudoalteromonas sp. GA327. *Antibiotics*, *9*(12), p.923. |
| 24 | 1-Undecanol | Garrido, A., Atencio, L.A., Bethancourt, R., Bethancourt, A., Guzmán, H., Gutiérrez, M. and Durant-Archibold, A.A., 2020. Antibacterial Activity of Volatile Organic Compounds Produced by the Octocoral-Associated Bacteria Bacillus sp. BO53 and Pseudoalteromonas sp. GA327. *Antibiotics*, *9*(12), p.923. |
| 25 | 2 Camphene | Hamad Al-Mijalli, S., ELsharkawy, E. R., Abdallah, E. M., Hamed, M., El Omari, N., Mahmud, S., ... & Bouyahya, A. (2022). Determination of Volatile Compounds of Mentha piperita and Lavandula multifida and Investigation of Their Antibacterial, Antioxidant, and Antidiabetic Properties. *Evidence-Based Complementary and Alternative Medicine*, *2022*. |
| 26 | acetate | Hamad Al-Mijalli, S., ELsharkawy, E. R., Abdallah, E. M., Hamed, M., El Omari, N., Mahmud, S., ... & Bouyahya, A. (2022). Determination of Volatile Compounds of Mentha piperita and Lavandula multifida and Investigation of Their Antibacterial, Antioxidant, and Antidiabetic Properties. *Evidence-Based Complementary and Alternative Medicine*, *2022*. |
| 27 | *p*-Menth-8-en-3-ol | Hamad Al-Mijalli, S., ELsharkawy, E. R., Abdallah, E. M., Hamed, M., El Omari, N., Mahmud, S., ... & Bouyahya, A. (2022). Determination of Volatile Compounds of Mentha piperita and Lavandula multifida and Investigation of Their Antibacterial, Antioxidant, and Antidiabetic Properties. *Evidence-Based Complementary and Alternative Medicine*, *2022*. |
| 28 | Alloaromadendrene | Hamad Al-Mijalli, S., ELsharkawy, E. R., Abdallah, E. M., Hamed, M., El Omari, N., Mahmud, S., ... & Bouyahya, A. (2022). Determination of Volatile Compounds of Mentha piperita and Lavandula multifida and Investigation of Their Antibacterial, Antioxidant, and Antidiabetic Properties. *Evidence-Based Complementary and Alternative Medicine*, *2022*. |
| 29 | Cadinol | Hamad Al-Mijalli, S., ELsharkawy, E. R., Abdallah, E. M., Hamed, M., El Omari, N., Mahmud, S., ... & Bouyahya, A. (2022). Determination of Volatile Compounds of Mentha piperita and Lavandula multifida and Investigation of Their Antibacterial, Antioxidant, and Antidiabetic Properties. *Evidence-Based Complementary and Alternative Medicine*, *2022*. |
| 30 | Caryophyllene oxide | Hamad Al-Mijalli, S., ELsharkawy, E. R., Abdallah, E. M., Hamed, M., El Omari, N., Mahmud, S., ... & Bouyahya, A. (2022). Determination of Volatile Compounds of Mentha piperita and Lavandula multifida and Investigation of Their Antibacterial, Antioxidant, and Antidiabetic Properties. *Evidence-Based Complementary and Alternative Medicine*, *2022*. |
| 31 | Germacrene *D* | Hamad Al-Mijalli, S., ELsharkawy, E. R., Abdallah, E. M., Hamed, M., El Omari, N., Mahmud, S., ... & Bouyahya, A. (2022). Determination of Volatile Compounds of Mentha piperita and Lavandula multifida and Investigation of Their Antibacterial, Antioxidant, and Antidiabetic Properties. *Evidence-Based Complementary and Alternative Medicine*, *2022*. |
| 32 | Caryophyllene | Hamad Al-Mijalli, S., ELsharkawy, E. R., Abdallah, E. M., Hamed, M., El Omari, N., Mahmud, S., ... & Bouyahya, A. (2022). Determination of Volatile Compounds of Mentha piperita and Lavandula multifida and Investigation of Their Antibacterial, Antioxidant, and Antidiabetic Properties. *Evidence-Based Complementary and Alternative Medicine*, *2022*. |
| 33 | Elemene | Hamad Al-Mijalli, S., ELsharkawy, E. R., Abdallah, E. M., Hamed, M., El Omari, N., Mahmud, S., ... & Bouyahya, A. (2022). Determination of Volatile Compounds of Mentha piperita and Lavandula multifida and Investigation of Their Antibacterial, Antioxidant, and Antidiabetic Properties. *Evidence-Based Complementary and Alternative Medicine*, *2022*. |
| 34 | Lavandulyl acetate | Hamad Al-Mijalli, S., ELsharkawy, E. R., Abdallah, E. M., Hamed, M., El Omari, N., Mahmud, S., ... & Bouyahya, A. (2022). Determination of Volatile Compounds of Mentha piperita and Lavandula multifida and Investigation of Their Antibacterial, Antioxidant, and Antidiabetic Properties. *Evidence-Based Complementary and Alternative Medicine*, *2022*. |
| 35 | Bornyl acetate | Hamad Al-Mijalli, S., ELsharkawy, E. R., Abdallah, E. M., Hamed, M., El Omari, N., Mahmud, S., ... & Bouyahya, A. (2022). Determination of Volatile Compounds of Mentha piperita and Lavandula multifida and Investigation of Their Antibacterial, Antioxidant, and Antidiabetic Properties. *Evidence-Based Complementary and Alternative Medicine*, *2022*. |
| 36 | Linalyl acetate | Hamad Al-Mijalli, S., ELsharkawy, E. R., Abdallah, E. M., Hamed, M., El Omari, N., Mahmud, S., ... & Bouyahya, A. (2022). Determination of Volatile Compounds of Mentha piperita and Lavandula multifida and Investigation of Their Antibacterial, Antioxidant, and Antidiabetic Properties. *Evidence-Based Complementary and Alternative Medicine*, *2022*. |
| 37 | *D*-Carvone | Hamad Al-Mijalli, S., ELsharkawy, E. R., Abdallah, E. M., Hamed, M., El Omari, N., Mahmud, S., ... & Bouyahya, A. (2022). Determination of Volatile Compounds of Mentha piperita and Lavandula multifida and Investigation of Their Antibacterial, Antioxidant, and Antidiabetic Properties. *Evidence-Based Complementary and Alternative Medicine*, *2022*. |
| 38 | Caren-2-ol | Hamad Al-Mijalli, S., ELsharkawy, E. R., Abdallah, E. M., Hamed, M., El Omari, N., Mahmud, S., ... & Bouyahya, A. (2022). Determination of Volatile Compounds of Mentha piperita and Lavandula multifida and Investigation of Their Antibacterial, Antioxidant, and Antidiabetic Properties. *Evidence-Based Complementary and Alternative Medicine*, *2022*. |
| 39 | Levomenthol | Hamad Al-Mijalli, S., ELsharkawy, E. R., Abdallah, E. M., Hamed, M., El Omari, N., Mahmud, S., ... & Bouyahya, A. (2022). Determination of Volatile Compounds of Mentha piperita and Lavandula multifida and Investigation of Their Antibacterial, Antioxidant, and Antidiabetic Properties. *Evidence-Based Complementary and Alternative Medicine*, *2022*. |
| 40 | Menthol | Hamad Al-Mijalli, S., ELsharkawy, E. R., Abdallah, E. M., Hamed, M., El Omari, N., Mahmud, S., ... & Bouyahya, A. (2022). Determination of Volatile Compounds of Mentha piperita and Lavandula multifida and Investigation of Their Antibacterial, Antioxidant, and Antidiabetic Properties. *Evidence-Based Complementary and Alternative Medicine*, *2022*. |
| 41 | Endo-borneol | Hamad Al-Mijalli, S., ELsharkawy, E. R., Abdallah, E. M., Hamed, M., El Omari, N., Mahmud, S., ... & Bouyahya, A. (2022). Determination of Volatile Compounds of Mentha piperita and Lavandula multifida and Investigation of Their Antibacterial, Antioxidant, and Antidiabetic Properties. *Evidence-Based Complementary and Alternative Medicine*, *2022*. |
| 42 | Menthone | Hamad Al-Mijalli, S., ELsharkawy, E. R., Abdallah, E. M., Hamed, M., El Omari, N., Mahmud, S., ... & Bouyahya, A. (2022). Determination of Volatile Compounds of Mentha piperita and Lavandula multifida and Investigation of Their Antibacterial, Antioxidant, and Antidiabetic Properties. *Evidence-Based Complementary and Alternative Medicine*, *2022*. |
| 43 | 2-Bornanone | Hamad Al-Mijalli, S., ELsharkawy, E. R., Abdallah, E. M., Hamed, M., El Omari, N., Mahmud, S., ... & Bouyahya, A. (2022). Determination of Volatile Compounds of Mentha piperita and Lavandula multifida and Investigation of Their Antibacterial, Antioxidant, and Antidiabetic Properties. *Evidence-Based Complementary and Alternative Medicine*, *2022*. |
| 44 | Linalool | Hamad Al-Mijalli, S., ELsharkawy, E. R., Abdallah, E. M., Hamed, M., El Omari, N., Mahmud, S., ... & Bouyahya, A. (2022). Determination of Volatile Compounds of Mentha piperita and Lavandula multifida and Investigation of Their Antibacterial, Antioxidant, and Antidiabetic Properties. *Evidence-Based Complementary and Alternative Medicine*, *2022*. |
| 45 | Terpinen-4-ol | Hamad Al-Mijalli, S., ELsharkawy, E. R., Abdallah, E. M., Hamed, M., El Omari, N., Mahmud, S., ... & Bouyahya, A. (2022). Determination of Volatile Compounds of Mentha piperita and Lavandula multifida and Investigation of Their Antibacterial, Antioxidant, and Antidiabetic Properties. *Evidence-Based Complementary and Alternative Medicine*, *2022*. |
| 46 | Eucalyptol | Hamad Al-Mijalli, S., ELsharkawy, E. R., Abdallah, E. M., Hamed, M., El Omari, N., Mahmud, S., ... & Bouyahya, A. (2022). Determination of Volatile Compounds of Mentha piperita and Lavandula multifida and Investigation of Their Antibacterial, Antioxidant, and Antidiabetic Properties. *Evidence-Based Complementary and Alternative Medicine*, *2022*. |
| 47 | 2,3-Dehydro-1,8-cineole | Hamad Al-Mijalli, S., ELsharkawy, E. R., Abdallah, E. M., Hamed, M., El Omari, N., Mahmud, S., ... & Bouyahya, A. (2022). Determination of Volatile Compounds of Mentha piperita and Lavandula multifida and Investigation of Their Antibacterial, Antioxidant, and Antidiabetic Properties. *Evidence-Based Complementary and Alternative Medicine*, *2022*. |
| 48 | 3-Carene | Hamad Al-Mijalli, S., ELsharkawy, E. R., Abdallah, E. M., Hamed, M., El Omari, N., Mahmud, S., ... & Bouyahya, A. (2022). Determination of Volatile Compounds of Mentha piperita and Lavandula multifida and Investigation of Their Antibacterial, Antioxidant, and Antidiabetic Properties. *Evidence-Based Complementary and Alternative Medicine*, *2022*. |
| 49 | dimethyl sulfide | Ossowicki, A., Jafra, S., & Garbeva, P. (2017). The antimicrobial volatile power of the rhizospheric isolate Pseudomonas donghuensis P482. *PloS one*, *12*(3), e0174362. |
| 50 | S-methyl thioacetate | Ossowicki, A., Jafra, S., & Garbeva, P. (2017). The antimicrobial volatile power of the rhizospheric isolate Pseudomonas donghuensis P482. *PloS one*, *12*(3), e0174362. |
| 51 | methyl thiocyanate | Ossowicki, A., Jafra, S., & Garbeva, P. (2017). The antimicrobial volatile power of the rhizospheric isolate Pseudomonas donghuensis P482. *PloS one*, *12*(3), e0174362. |
| 52 | dimethyl trisulfide | Ossowicki, A., Jafra, S., & Garbeva, P. (2017). The antimicrobial volatile power of the rhizospheric isolate Pseudomonas donghuensis P482. *PloS one*, *12*(3), e0174362. |
| 53 | 1-undecan | Ossowicki, A., Jafra, S., & Garbeva, P. (2017). The antimicrobial volatile power of the rhizospheric isolate Pseudomonas donghuensis P482. *PloS one*, *12*(3), e0174362. |
| 54 | hydrogen cyanide | Ossowicki, A., Jafra, S., & Garbeva, P. (2017). The antimicrobial volatile power of the rhizospheric isolate Pseudomonas donghuensis P482. *PloS one*, *12*(3), e0174362. |
| 55 | Palmitic acid | Xie, Y., Peng, Q., Ji, Y., Xie, A., Yang, L., Mu, S., ... & Zhang, Q. (2021). Isolation and identification of antibacterial bioactive compounds from Bacillus megaterium L2. *Frontiers in microbiology*, *12*, 645484. |
| 56 | Phenylacetic acid | Xie, Y., Peng, Q., Ji, Y., Xie, A., Yang, L., Mu, S., ... & Zhang, Q. (2021). Isolation and identification of antibacterial bioactive compounds from Bacillus megaterium L2. *Frontiers in microbiology*, *12*, 645484. |
| 57 | b-sitosterol | Xie, Y., Peng, Q., Ji, Y., Xie, A., Yang, L., Mu, S., ... & Zhang, Q. (2021). Isolation and identification of antibacterial bioactive compounds from Bacillus megaterium L2. *Frontiers in microbiology*, *12*, 645484. |
| 58 | 1-Heptanal | Lammers, A., Zweers, H., Sandfeld, T., Bilde, T., Garbeva, P., Schramm, A., & Lalk, M. (2021). Antimicrobial compounds in the volatilome of social spider communities. *Frontiers in microbiology*, *12*. |
| 59 | 2-Ethylhexanol | Lammers, A., Zweers, H., Sandfeld, T., Bilde, T., Garbeva, P., Schramm, A., & Lalk, M. (2021). Antimicrobial compounds in the volatilome of social spider communities. *Frontiers in microbiology*, *12*. |
| 60 | Acetophenone | Lammers, A., Zweers, H., Sandfeld, T., Bilde, T., Garbeva, P., Schramm, A., & Lalk, M. (2021). Antimicrobial compounds in the volatilome of social spider communities. *Frontiers in microbiology*, *12*. |
| 61 | Dodecane | Lammers, A., Zweers, H., Sandfeld, T., Bilde, T., Garbeva, P., Schramm, A., & Lalk, M. (2021). Antimicrobial compounds in the volatilome of social spider communities. *Frontiers in microbiology*, *12*. |
| 62 | 1-Decanal | Lammers, A., Zweers, H., Sandfeld, T., Bilde, T., Garbeva, P., Schramm, A., & Lalk, M. (2021). Antimicrobial compounds in the volatilome of social spider communities. *Frontiers in microbiology*, *12*. |
| 63 | 2-methylpropanoate | Lammers, A., Zweers, H., Sandfeld, T., Bilde, T., Garbeva, P., Schramm, A., & Lalk, M. (2021). Antimicrobial compounds in the volatilome of social spider communities. *Frontiers in microbiology*, *12*. |
| 64 | 1-Tetradecene | Lammers, A., Zweers, H., Sandfeld, T., Bilde, T., Garbeva, P., Schramm, A., & Lalk, M. (2021). Antimicrobial compounds in the volatilome of social spider communities. *Frontiers in microbiology*, *12*. |
| 65 | 1-Dodecanal | Lammers, A., Zweers, H., Sandfeld, T., Bilde, T., Garbeva, P., Schramm, A., & Lalk, M. (2021). Antimicrobial compounds in the volatilome of social spider communities. *Frontiers in microbiology*, *12*. |
| 66 | Myristic acid | Lammers, A., Zweers, H., Sandfeld, T., Bilde, T., Garbeva, P., Schramm, A., & Lalk, M. (2021). Antimicrobial compounds in the volatilome of social spider communities. *Frontiers in microbiology*, *12*. |
| 67 | Pentadecane | Lammers, A., Zweers, H., Sandfeld, T., Bilde, T., Garbeva, P., Schramm, A., & Lalk, M. (2021). Antimicrobial compounds in the volatilome of social spider communities. *Frontiers in microbiology*, *12*. |
| 68 | Nerylacetone | Lammers, A., Zweers, H., Sandfeld, T., Bilde, T., Garbeva, P., Schramm, A., & Lalk, M. (2021). Antimicrobial compounds in the volatilome of social spider communities. *Frontiers in microbiology*, *12*. |
| 69 | 1-Dodecanol | Lammers, A., Zweers, H., Sandfeld, T., Bilde, T., Garbeva, P., Schramm, A., & Lalk, M. (2021). Antimicrobial compounds in the volatilome of social spider communities. *Frontiers in microbiology*, *12*. |
| 70 | 2-Ethyl-3-hydroxyhexyl | Lammers, A., Zweers, H., Sandfeld, T., Bilde, T., Garbeva, P., Schramm, A., & Lalk, M. (2021). Antimicrobial compounds in the volatilome of social spider communities. *Frontiers in microbiology*, *12*. |
| 71 | 1,3-Benzothiazole | Lammers, A., Zweers, H., Sandfeld, T., Bilde, T., Garbeva, P., Schramm, A., & Lalk, M. (2021). Antimicrobial compounds in the volatilome of social spider communities. *Frontiers in microbiology*, *12*. |
| 72 | 1-Undecanol | Lammers, A., Zweers, H., Sandfeld, T., Bilde, T., Garbeva, P., Schramm, A., & Lalk, M. (2021). Antimicrobial compounds in the volatilome of social spider communities. *Frontiers in microbiology*, *12*. |
| 73 | 1-Dodecene | Lammers, A., Zweers, H., Sandfeld, T., Bilde, T., Garbeva, P., Schramm, A., & Lalk, M. (2021). Antimicrobial compounds in the volatilome of social spider communities. *Frontiers in microbiology*, *12*. |
| 74 | 1-Tridecene | Lammers, A., Zweers, H., Sandfeld, T., Bilde, T., Garbeva, P., Schramm, A., & Lalk, M. (2021). Antimicrobial compounds in the volatilome of social spider communities. *Frontiers in microbiology*, *12*. |
| 75 | 2-nonanone | Raza, W., Ling, N., Yang, L., Huang, Q., & Shen, Q. (2016). Response of tomato wilt pathogen Ralstonia solanacearum to the volatile organic compounds produced by a biocontrol strain Bacillus amyloliquefaciens SQR-9. *Scientific reports*, *6*(1), 1-13. |
| 76 | 2-undecanone | Raza, W., Ling, N., Yang, L., Huang, Q., & Shen, Q. (2016). Response of tomato wilt pathogen Ralstonia solanacearum to the volatile organic compounds produced by a biocontrol strain Bacillus amyloliquefaciens SQR-9. *Scientific reports*, *6*(1), 1-13. |
| 77 | 2-dodecanone | Raza, W., Ling, N., Yang, L., Huang, Q., & Shen, Q. (2016). Response of tomato wilt pathogen Ralstonia solanacearum to the volatile organic compounds produced by a biocontrol strain Bacillus amyloliquefaciens SQR-9. *Scientific reports*, *6*(1), 1-13. |
| 78 | 2-tridecanone | Raza, W., Ling, N., Yang, L., Huang, Q., & Shen, Q. (2016). Response of tomato wilt pathogen Ralstonia solanacearum to the volatile organic compounds produced by a biocontrol strain Bacillus amyloliquefaciens SQR-9. *Scientific reports*, *6*(1), 1-13. |
| 79 | 2-tetradecanone | Raza, W., Ling, N., Yang, L., Huang, Q., & Shen, Q. (2016). Response of tomato wilt pathogen Ralstonia solanacearum to the volatile organic compounds produced by a biocontrol strain Bacillus amyloliquefaciens SQR-9. *Scientific reports*, *6*(1), 1-13. |
| 80 | 2-pentadecanone | Raza, W., Ling, N., Yang, L., Huang, Q., & Shen, Q. (2016). Response of tomato wilt pathogen Ralstonia solanacearum to the volatile organic compounds produced by a biocontrol strain Bacillus amyloliquefaciens SQR-9. *Scientific reports*, *6*(1), 1-13. |
| 81 | Hexadecanal | Raza, W., Ling, N., Yang, L., Huang, Q., & Shen, Q. (2016). Response of tomato wilt pathogen Ralstonia solanacearum to the volatile organic compounds produced by a biocontrol strain Bacillus amyloliquefaciens SQR-9. *Scientific reports*, *6*(1), 1-13. |
| 82 | Undecanal | Raza, W., Ling, N., Yang, L., Huang, Q., & Shen, Q. (2016). Response of tomato wilt pathogen Ralstonia solanacearum to the volatile organic compounds produced by a biocontrol strain Bacillus amyloliquefaciens SQR-9. *Scientific reports*, *6*(1), 1-13. |
| 83 | Nonanal | Raza, W., Ling, N., Yang, L., Huang, Q., & Shen, Q. (2016). Response of tomato wilt pathogen Ralstonia solanacearum to the volatile organic compounds produced by a biocontrol strain Bacillus amyloliquefaciens SQR-9. *Scientific reports*, *6*(1), 1-13. |
| 84 | Tridecane | Raza, W., Ling, N., Yang, L., Huang, Q., & Shen, Q. (2016). Response of tomato wilt pathogen Ralstonia solanacearum to the volatile organic compounds produced by a biocontrol strain Bacillus amyloliquefaciens SQR-9. *Scientific reports*, *6*(1), 1-13. |
| 85 | Heptadecane | Raza, W., Ling, N., Yang, L., Huang, Q., & Shen, Q. (2016). Response of tomato wilt pathogen Ralstonia solanacearum to the volatile organic compounds produced by a biocontrol strain Bacillus amyloliquefaciens SQR-9. *Scientific reports*, *6*(1), 1-13. |
| 86 | Furan 2-ethyl-5-methyl | Raza, W., Ling, N., Yang, L., Huang, Q., & Shen, Q. (2016). Response of tomato wilt pathogen Ralstonia solanacearum to the volatile organic compounds produced by a biocontrol strain Bacillus amyloliquefaciens SQR-9. *Scientific reports*, *6*(1), 1-13. |
| 87 | Phenylethyl alcohol | Raza, W., Ling, N., Yang, L., Huang, Q., & Shen, Q. (2016). Response of tomato wilt pathogen Ralstonia solanacearum to the volatile organic compounds produced by a biocontrol strain Bacillus amyloliquefaciens SQR-9. *Scientific reports*, *6*(1), 1-13. |
| 88 | Oleic acid | Raza, W., Ling, N., Yang, L., Huang, Q., & Shen, Q. (2016). Response of tomato wilt pathogen Ralstonia solanacearum to the volatile organic compounds produced by a biocontrol strain Bacillus amyloliquefaciens SQR-9. *Scientific reports*, *6*(1), 1-13. |
| 89 | Hexadecanoic acid | Raza, W., Ling, N., Yang, L., Huang, Q., & Shen, Q. (2016). Response of tomato wilt pathogen Ralstonia solanacearum to the volatile organic compounds produced by a biocontrol strain Bacillus amyloliquefaciens SQR-9. *Scientific reports*, *6*(1), 1-13. |
| 90 | n-hexanoic acid | Raza, W., Ling, N., Yang, L., Huang, Q., & Shen, Q. (2016). Response of tomato wilt pathogen Ralstonia solanacearum to the volatile organic compounds produced by a biocontrol strain Bacillus amyloliquefaciens SQR-9. *Scientific reports*, *6*(1), 1-13. |
| 91 | 1-Hexanol, 2-ethyl- | Che, J., Liu, B., Liu, G., Chen, Q., & Lan, J. (2017). Volatile organic compounds produced by Lysinibacillus sp. FJAT-4748 possess antifungal activity against Colletotrichum acutatum. *Biocontrol Science and Technology*, *27*(12), 1349-1362. |
| 92 | Piperidine, 1-ethyl- | Che, J., Liu, B., Liu, G., Chen, Q., & Lan, J. (2017). Volatile organic compounds produced by Lysinibacillus sp. FJAT-4748 possess antifungal activity against Colletotrichum acutatum. *Biocontrol Science and Technology*, *27*(12), 1349-1362. |
| 93 | Benzaldehyde | Che, J., Liu, B., Liu, G., Chen, Q., & Lan, J. (2017). Volatile organic compounds produced by Lysinibacillus sp. FJAT-4748 possess antifungal activity against Colletotrichum acutatum. *Biocontrol Science and Technology*, *27*(12), 1349-1362. |
| 94 | Rhizocticin-a | Tran, C., Cock, I. E., Chen, X., & Feng, Y. (2022). Antimicrobial Bacillus: Metabolites and Their Mode of Action. *Antibiotics*, *11*(1), 88. |
| 95 | Macrolactin-n | Tran, C., Cock, I. E., Chen, X., & Feng, Y. (2022). Antimicrobial Bacillus: Metabolites and Their Mode of Action. *Antibiotics*, *11*(1), 88. |
| 96 | promycin | Tran, C., Cock, I. E., Chen, X., & Feng, Y. (2022). Antimicrobial Bacillus: Metabolites and Their Mode of Action. *Antibiotics*, *11*(1), 88. |
| 97 | chlorotetaine | Tran, C., Cock, I. E., Chen, X., & Feng, Y. (2022). Antimicrobial Bacillus: Metabolites and Their Mode of Action. *Antibiotics*, *11*(1), 88. |
| 98 | Bacilysin | Tran, C., Cock, I. E., Chen, X., & Feng, Y. (2022). Antimicrobial Bacillus: Metabolites and Their Mode of Action. *Antibiotics*, *11*(1), 88. |
| 99 | Azoxybacilin | Tran, C., Cock, I. E., Chen, X., & Feng, Y. (2022). Antimicrobial Bacillus: Metabolites and Their Mode of Action. *Antibiotics*, *11*(1), 88. |
| 100 | Stigmatellin | Tran, C., Cock, I. E., Chen, X., & Feng, Y. (2022). Antimicrobial Bacillus: Metabolites and Their Mode of Action. *Antibiotics*, *11*(1), 88. |

**Table S2A**. Virtual screening results of five best compounds against guaA.

1. Enfumafungin

mode | affinity | dist from best mode

| (kcal/mol) | rmsd l.b.| rmsd u.b.

-----+------------+----------+----------

1 -8.0 0.000 0.000

2 -7.6 4.706 10.308

3 -7.5 5.653 10.549

4 -7.2 11.588 14.497

5 -7.1 25.160 31.968

6 -7.1 6.950 11.070

7 -7.0 9.968 16.982

8 -7.0 10.327 13.866

9 -7.0 33.912 41.269

10 -7.0 42.106 48.747

2. Favolon

mode | affinity | dist from best mode

| (kcal/mol) | rmsd l.b.| rmsd u.b.

-----+------------+----------+----------

1 -8.1 0.000 0.000

2 -7.6 4.671 9.165

3 -7.5 22.771 25.622

4 -7.5 23.098 24.470

5 -7.3 33.631 37.094

6 -7.1 6.910 10.637

7 -7.1 7.366 13.211

8 -7.0 3.680 12.011

9 -6.9 4.066 11.786

10 -6.9 31.980 34.689

3. Macrolactine. N

mode | affinity | dist from best mode

| (kcal/mol) | rmsd l.b.| rmsd u.b.

-----+------------+----------+----------

1 -8.3 0.000 0.000

2 -8.1 2.131 7.884

3 -7.8 10.654 14.425

4 -7.5 4.062 8.231

5 -7.4 2.263 5.140

6 -7.3 52.372 56.673

7 -7.2 51.424 54.757

8 -7.2 3.521 8.731

9 -7.2 50.863 55.705

10 -7.0 36.353 38.867

4. Rubrolide.S

mode | affinity | dist from best mode

| (kcal/mol) | rmsd l.b.| rmsd u.b.

-----+------------+----------+----------

1 -8.7 0.000 0.000

2 -8.5 47.001 53.001

3 -8.3 2.560 3.963

4 -8.0 38.413 43.356

5 -7.8 4.301 6.718

6 -7.8 43.688 46.655

7 -7.7 50.758 55.756

8 -7.7 46.630 51.541

9 -7.6 24.299 26.823

10 -7.5 44.177 49.616

5. Trypilepyrazinol.

mode | affinity | dist from best mode

| (kcal/mol) | rmsd l.b.| rmsd u.b.

-----+------------+----------+----------

1 -8.0 0.000 0.000

2 -7.2 40.499 45.026

3 -7.1 4.770 6.619

4 -6.5 4.172 6.581

5 -6.2 40.994 44.761

6 -6.1 9.183 12.063

7 -6.0 41.823 43.411

8 -6.0 23.474 25.356

9 -5.9 5.801 7.696

10 -5.8 41.441 45.856

**Table S2 B.** Virtual screening results of five best compounds against metG.

1. B.sitosterol.

mode | affinity | dist from best mode

| (kcal/mol) | rmsd l.b.| rmsd u.b.

-----+------------+----------+----------

1 -8.6 0.000 0.000

2 -8.5 1.603 2.908

3 -8.2 54.745 60.353

4 -8.1 33.846 35.073

5 -8.0 33.741 35.107

6 -7.8 5.193 9.193

7 -7.7 5.263 8.466

8 -7.5 54.871 60.482

9 -7.4 3.793 7.029

10 -7.4 64.544 68.001

2. Enfumafungin.

mode | affinity | dist from best mode

| (kcal/mol) | rmsd l.b.| rmsd u.b.

-----+------------+----------+----------

1 -8.1 0.000 0.000

2 -7.9 47.074 50.201

3 -7.8 6.324 7.709

4 -7.8 2.767 5.456

5 -7.7 54.657 59.807

6 -7.7 6.963 9.510

7 -7.4 10.798 13.899

8 -7.2 13.070 16.095

9 -7.2 47.150 50.247

10 -7.2 16.186 19.901

3. Favolon.

mode | affinity | dist from best mode

| (kcal/mol) | rmsd l.b.| rmsd u.b.

-----+------------+----------+----------

1 -8.8 0.000 0.000

2 -8.8 1.586 2.246

3 -8.8 1.524 2.730

4 -8.7 60.979 63.892

5 -8.6 61.105 64.009

6 -8.6 60.930 63.856

7 -8.4 16.652 18.369

8 -8.2 54.110 57.246

9 -8.1 19.746 22.303

10 -8.1 19.448 23.304

4. macrolactin

mode | affinity | dist from best mode

| (kcal/mol) | rmsd l.b.| rmsd u.b.

-----+------------+----------+----------

1 -8.9 0.000 0.000

2 -8.2 2.825 7.182

3 -8.1 29.792 33.255

4 -8.1 1.956 2.696

5 -8.1 50.355 54.189

6 -8.0 56.576 60.273

7 -7.7 57.649 61.373

8 -7.6 56.296 60.085

9 -7.6 25.164 28.634

10 -7.5 57.599 61.131

5. Neihumicin.

mode | affinity | dist from best mode

| (kcal/mol) | rmsd l.b.| rmsd u.b.

-----+------------+----------+----------

1 -7.8 0.000 0.000

2 -7.7 1.250 8.165

3 -7.6 4.822 10.408

4 -7.5 2.437 3.926

5 -7.3 28.601 31.664

6 -7.2 4.012 6.154

7 -7.1 56.743 58.830

8 -7.1 57.117 58.648

9 -7.1 6.123 9.270

10 -7.0 19.110 21.786

**Table S3 A.** Top 10 ranking proteins in Closeness, Betweenness, Eigenvector and Degree centrality in descending order.

| Gene name | Closeness | Gene  name | Betweenness | Gene  name | Eigenvector | Gene name | Degree |
| --- | --- | --- | --- | --- | --- | --- | --- |
| guaA | 0.428571 | \| purL \| \| --- \| | 0.186994522 | infB | 0.28194 | guaA | 120 |
| purL | 0.407529 | guaA | 0.176596895 | metG | 0.231332 | metG | 100 |
| metG | 0.402913 | bamA | 0.110797211 | rplB | 0.221399 | purL | 84 |
| glyA | 0.395866 | ftsZ | 0.106014291 | rpsC | 0.217659 | glyA | 78 |
| tpiA | 0.373313 | metG | 0.102059962 | rplD | 0.21122 | rpsC | 70 |
| rpsC | 0.373313 | yidC | 0.097821877 | rplC | 0.211052 | rplB | 68 |
| pyrG | 0.361919 | lptD | 0.090765141 | lysS | 0.20879 | rplD | 66 |
| purD | 0.361919 | glyA | 0.089555034 | rpsG | 0.208467 | rplV | 62 |
| ileS | 0.361393 | lysS | 0.08255055 | guaA | 0.205405 | rpsG | 62 |
| rplB | 0.36087 | hisD | 0.077335 | rplV | 0.199183 | rplC | 62 |

**Table S3 B****:** Common proteins in each category, the red colored proteins are common in all categories such as Closeness", "Betweenness", "Eigenvector" and "Degree".

| 2 common elements in "Closeness", "Betweenness", "Eigenvector" and "Degree": | 2 common elements in "Closeness", "Eigenvector" and "Degree": | 2 common elements in "Closeness", "Betweenness" and "Degree": | 4 common elements in "Eigenvector" and "Degree": |
| --- | --- | --- | --- |
| guaA | rpsC | purL | rplD |
| metG | rplB | glyA | rplC |
|  |  |  | rpsG |
|  |  |  | rplV |
